# Anatomy- and Time-Based Post-Processing with CT Perfusion-Derived Time–Vessel Maps to Reduce Incorrect Occlusion Detections in Late-Phase CT Angiography

**DOI:** 10.3390/diagnostics16182972

**Published:** 2026-09-14

**Authors:** Andrés Martínez Mora, Mahsa Mojtahedi, Lucas de Vries, Michael Baumgartner, Yannick Kirchhoff, Maximilian Zenk, Katharina Eckstein, Jessica Kächele, Gianluca Brugnara, Martin Bendszus, Alexander Radbruch, Yvo Roos, Wim van Zwam, Robert van Oostenbrugge, Diederik W. J. Dippel, Bart J. Emmer, Charles Majoie, Philipp Vollmuth, Clara I. Sánchez, Klaus H. Maier-Hein, Henk A. Marquering

**Affiliations:** 1 Department of Medical Image Computing, German Cancer Research Center, 69120 Heidelberg, Germany; 2 Department of Radiology and Nuclear Medicine, Amsterdam University Medical Centers, Location AMC, 1105 AZ Amsterdam, The Netherlands; 3 Department of Biomedical Engineering and Physics, Amsterdam University Medical Centers, Location AMC, 1105 AZ Amsterdam, The Netherlands; 4 Quantitative Healthcare Analysis (QurAI) Group, Informatics Institute, University of Amsterdam, 1098 XH Amsterdam, The Netherlands; 5 Medical Faculty Heidelberg, University of Heidelberg, 69120 Heidelberg, Germany; 6 Konrad Zuse School of Excellence in Learning and Intelligent Systems (ELIZA), Technical University of Darmstadt, 64289 Darmstadt, Germany; 7 Amsterdam Cardiovascular Sciences, 1105 AZ Amsterdam, The Netherlands; 8 Amsterdam Neuroscience, 1105 AZ Amsterdam, The Netherlands; 9 Helmholtz Imaging, German Cancer Research Center, 69120 Heidelberg, Germany; 10 Faculty of Mathematics and Computer Science, University of Heidelberg, 69120 Heidelberg, Germany; 11 HIDSS4Health—Helmholtz Information and Data Science School for Health, Karlsruhe Institute of Technology, 76131 Karlsruhe, Germany; 12 HIDSS4Health—Helmholtz Information and Data Science School for Health, Karlsruhe Institute of Technology, 69120 Heidelberg, Germany; 13 German Cancer Consortium (DKTK), German Cancer Research Center (DKFZ), Core Center Heidelberg, 69120 Heidelberg, Germany; 14 Clinic for Neuroradiology, University Hospital Bonn, 53127 Bonn, Germany; 15 Department of Neuroradiology, Heidelberg University Hospital, 69120 Heidelberg, Germany; 16 Medical Faculty, University of Bonn, 53113 Bonn, Germany; 17 Department of Radiology and Nuclear Medicine, Maastricht University Medical Center, 6229 HX Maastricht, The Netherlands; 18 School for Cardiovascular Diseases (CARIM), Maastricht University, 6200 MD Maastricht, The Netherlands; 19 Department of Neurorology, Erasmus Medical Center, 3015 MD Rotterdam, The Netherlands; 20 National Center for Tumor Diseases (NCT), 69120 Heidelberg, Germany; 21 Pattern Analysis and Learning Group, Heidelberg University Hospital, 69120 Heidelberg, Germany

**Keywords:** acute ischemic stroke, CT angiography, CT perfusion, vessel occlusion detection, anatomical priors, temporal priors, incorrect occlusion removal

## Abstract

**Background/Objectives:** Computer-aided vessel occlusion detection for acute ischemic stroke is typically developed on early-phase CT angiography (CTA). In late-phase CTA, enhanced venous contrast can mimic arterial occlusions and lead to incorrect occlusion detections. To counteract this, we propose a constraint-based post-processing framework that leverages anatomical and temporal vascular information from CT perfusion (CTP) scans. **Methods:** Vascular anatomy and relative contrast-arrival times were encoded as time–vessel maps. Three complementary constraints worked on these maps to remove incorrect detections based on distance-to-vessel truncation points, contrast-arrival time, and spatial priors. Constraints were fixed on a 32-case late-phase development subset and applied to an external held-out cohort of late-phase CTA (*N* = 354). To enable application without CTP, an auxiliary CTA-to-time–vessel map generator was designed to estimate maps directly from CTA. **Results:** In the external cohort, the framework reduced incorrect occlusions per scan from 3.9 (95% CI 3.6–4.2) to 2.1 (95% CI 1.9–2.3), preserving occlusion-level sensitivity at 80% (95% CI 69–91). The patient-level AUROC was 83 for the baseline versus 88 with post-processing. Improvements were also observed in detectors exposed to late-phase CTA, where post-processing reduced incorrect occlusions per scan from 2.8 to 1.9. As only 51 occlusion-positive cases were present in the external cohort, confidence intervals were wide. Consequently, these estimates should be interpreted with caution. **Conclusions:** Anatomically and temporally informed post-processing reduced incorrect occlusion detections in late-phase CTA without measurable loss of sensitivity, potentially improving robustness against CTA phase changes in external institutions.

## 1. Introduction

**Figure 1:**
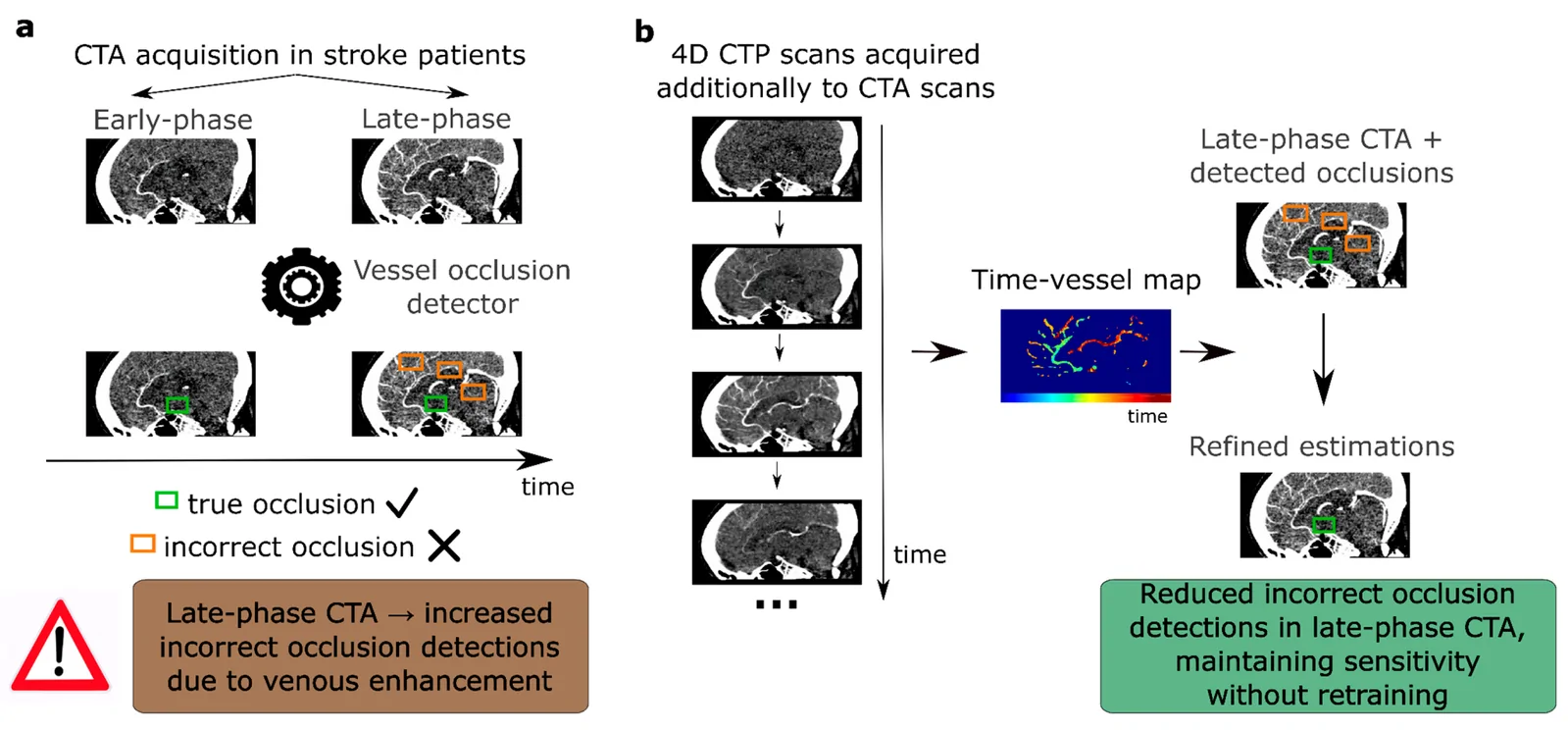
(**a**) CTA scans can be acquired in early- or late-phase. Venous enhancement in late-phase CTA can lead to incorrect occlusion detections from automated vessel occlusion detectors. (**b**) The proposed approach removes incorrect occlusion detections leveraging vascular anatomy and contrast-arrival time from CTP as time-vessel maps. Time-vessel maps represent vessels with their contrast arrival times, depicted here with a jet colormap. CTA, CT angiography; CTP, CT perfusion.

Acute ischemic stroke (AIS) is generally caused by occlusion of intracranial arteries and requires rapid and accurate diagnosis to enable timely reperfusion treatment. CT angiography (CTA) is widely used to identify vessel occlusions by visualizing the cerebrovascular tree after intravenous contrast administration [1]. Since manual vessel occlusion analysis is time-consuming and operator-dependent, computer-aided approaches have been developed to improve efficiency. Early automated approaches focused on binary classification of occlusion presence without estimating precise occlusion location [2,3,4,5,6,7], while subsequent methods addressed localization of large vessel occlusions [8,9]. Most recently, contributions in the field have extended to medium-vessel occlusions [10]. These methods are mostly developed on CTA scans in the early or peak arterial phase, when arterial contrast enhancement is maximal and vessel occlusions can be more easily detected [11]. In routine practice, however, CTA may be acquired in equilibrium or venous phases due to workflow variability or protocol differences, resulting in increased venous enhancement and reduced arterial enhancement [12]. In such delayed acquisitions, enhanced veins can mimic the appearance of arterial occlusions and thus increase the risk of incorrect detections by automated approaches. Delayed acquisitions remain a relevant challenge, since CTA scans in the equilibrium or venous phase may account for approximately 10% of CTA scans, particularly in non-specialized stroke centers [13]. As a result, when computer-aided systems are applied to late-phase CTA scans from other institutions, the increased venous enhancement can lead to incorrect occlusion detections, as illustrated in Figure 1a.

A possible way to identify implausible occlusions from computer-aided systems in late-phase scans is by using prior anatomical and temporal vascular information from multiphase data. Prior studies have shown that anatomical and temporal information from multiphase image modalities such as CTP and 4D CTA aid occlusion assessment by improving human rater performance [14,15], characterizing delayed vascular filling [16], supporting treatment selection [17], or improving medium vessel occlusion detection [18]. Multiphase information has also been incorporated into automated systems, either through direct use of multiphase inputs [19,20] or through intermediate anatomical and temporal representations [21]. These approaches, however, have not specifically addressed the suppression of incorrect occlusion detections in late-phase CTA.

In this work, we propose a constraint-based post-processing pipeline to improve the robustness of computer-aided vessel occlusion detection in the delayed acquisition phase. To provide the post-processing pipeline with relevant vascular information, we extract anatomical and temporal information from CTP scans that identifies implausible occlusion detections based on distance to truncated vessels, location in late contrast enhancement regions, and occlusion location in specific cerebral regions. A graphical summary of the proposed pipeline is included in Figure 1b. To enable application in patients without CTP, we further introduce an auxiliary generator that estimates vascular anatomy and relative contrast-arrival time directly from CTA. Rather than claiming improved raw detection, we specifically target a reduction in incorrect occlusion detections at preserved sensitivity. We evaluate the framework on an independent multicenter cohort and show that it improves detection for a detector trained on early-phase data and for a detector including late-phase scans in the training set, termed “late-phase retraining” from now on.

## 2. Materials and Methods

This retrospective multicenter study was approved by the Institutional Review Boards of the participating centers (generator training cohort and late-phase development subset: W19_281 #19.334; detector training cohort and external test cohort: S784/2018). Written informed consent was waived owing to the study’s retrospective design. An AI-based assistant was used for language editing and formatting.

### 2.1. Overview

**Figure 2:**
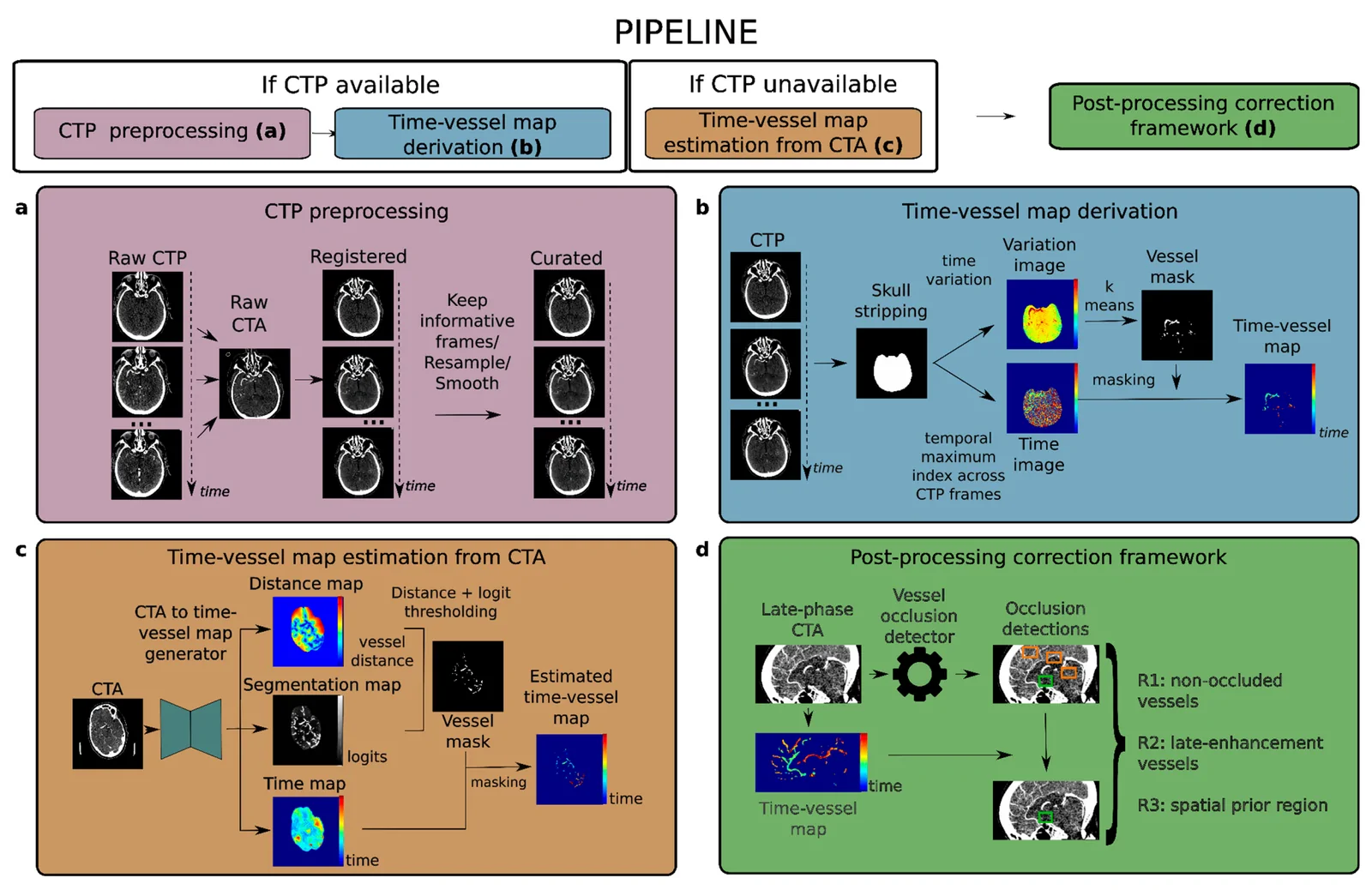
Pipeline for removing incorrect vessel-occlusion detections in late-phase CTA using perfusion-derived information. When CTP is available, CTP scans are preprocessed in (**a**), followed by time-vessel map extraction after computing vessel locations and times in (**b**). When CTP is unavailable, the time–vessel map is estimated with a CTA-to-time–vessel map generator in (**c**). In the end, a post-processing correction framework subsequently discards implausible occlusion detections (**d**). Colors represent different blocks of the pipeline. Full procedural detail is given in Section 2.5, Section 2.6, Section 2.7 and Section 2.8. CTA, CT angiography; CTP, CT perfusion.

We propose a constraint-based post-processing correction framework to reduce incorrect vessel occlusion detections in late-phase CTA, leveraging time-vessel maps from CTP scans. The overall pipeline consists of (1) a baseline occlusion detector, (2) the proposed constraint-based post-processing correction framework, and (3) an auxiliary CTA-to-time–vessel map generator that enables application in patients without CTP. A pipeline overview is presented in Figure 2. All pipeline components were implemented in Python 3.10. The code can be accessed in https://github.com/MIC-DKFZ/postprocessing_lateCTA (accessed on 14 August 2026).

### 2.2. Study Design, Patient Population, and Cohort Definitions

**Figure 3:**
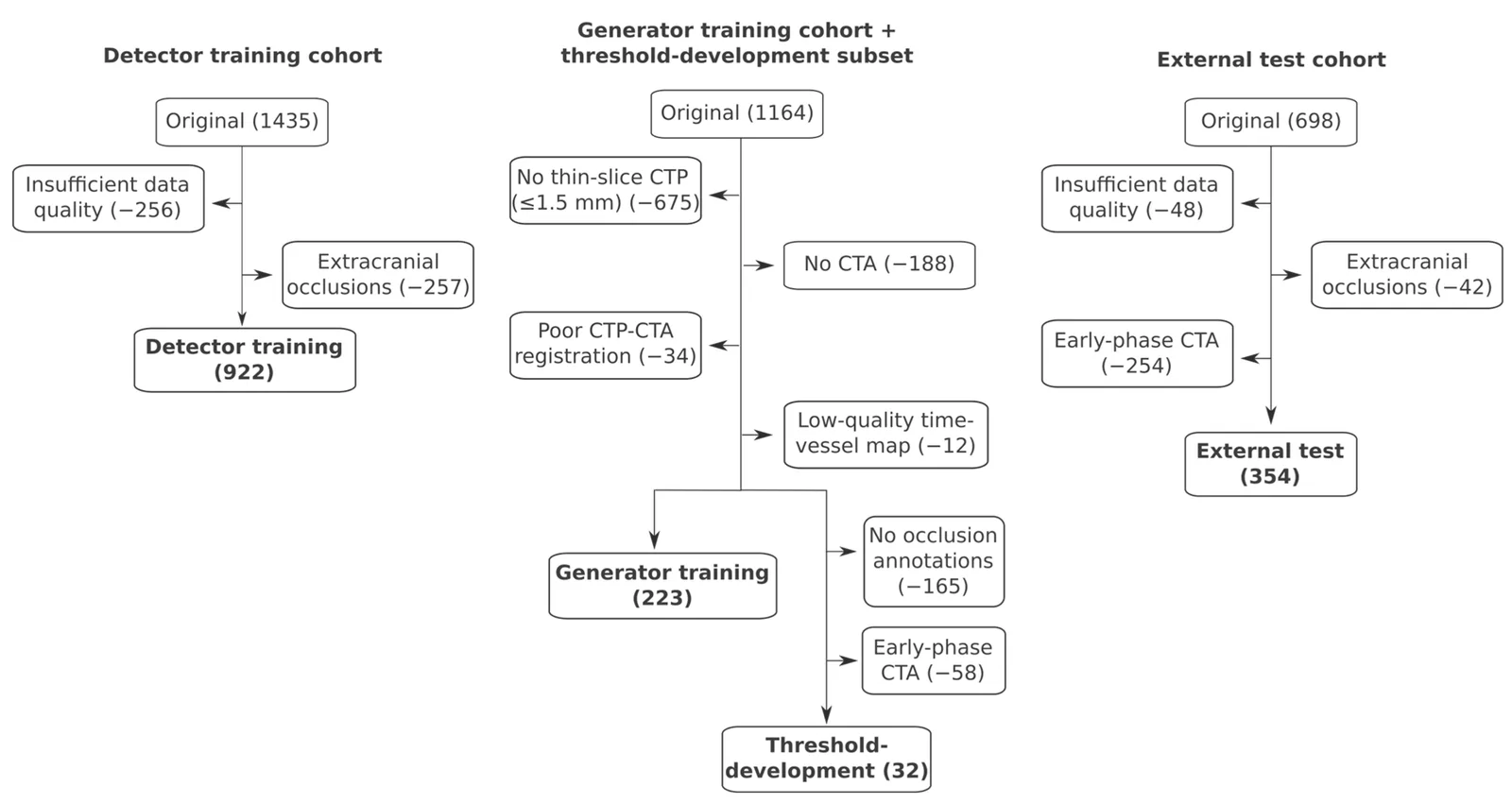
Exclusion criteria for the study data sets. CTA, CT angiography; CTP, CT perfusion.

Exclusion criteria for all datasets are summarized in Figure 3. To avoid ambiguity, we use function-based cohort names throughout the manuscript and figures, following the reviewers’ recommendation.

Detector training cohort: This cohort included AIS-suspected patients with CTA, including both occlusion-positive and occlusion-negative cases, predominantly early-phase, used exclusively to train the baseline detector. The scans were previously acquired for the development of vessel occlusion detectors [10]. 256 scans were excluded due to insufficient image quality, caused by lack of contrast, movement artifacts, or corrupted data. Extracranial occlusion cases were excluded from this cohort because they were not present by design on the CLEOPATRA dataset collection [16,22], the main data source for the generator training cohort and in the threshold-development subset.

Generator training cohort: This cohort comprised a multicenter AIS collection derived from the CLEOPATRA dataset, a collection of trials and a registry acquired to determine the value of CTP in stroke care. From the original dataset we gathered CTA volumes and thin-slice CTP scans with a slice thickness under 1.5 mm. All cases included intracranial occlusions and did not present any extracranial occlusions, per the design of the CLEOPATRA collection. This cohort was used to train the CTA-to-time–vessel map generator.

Threshold-development subset: This subject contained late-phase CTA cases with vessel occlusion annotations, obtained from the same source as the generator training cohort, used to determine post-processing framework parameters and to evaluate the generator. Post-processing parameters were frozen a priori and not modified in any subsequent analysis.

External test cohort: This cohort included patients with late-phase CTA from four centers with available vessel occlusion annotations but without paired CTP scans. The cohort included occlusion-positive and occlusion-negative cases and was used exclusively for independent evaluation [10]. The scans were originally acquired in routine clinical practice. A total of 48 scans were excluded due to insufficient image quality, caused by lack of contrast, movement artifacts, or corrupted data. As in the detector training cohort, extracranial occlusion cases were removed because they were not present in the generator training cohort and in the threshold-development subset.

The CTA phase for scans from all cohorts was assigned by expert annotators using the classification algorithm of Rodriguez-Luna et al., which compares Hounsfield units (HUs) in the middle cerebral artery to those in the venous sinuses [11]. Scans were sorted into early-arterial, peak-arterial, equilibrium, peak-venous, or late-venous phases. The first two categories (early-arterial, peak-arterial) were considered early-phase, with a higher arterial contrast than venous contrast, and the remaining categories (equilibrium, peak-venous, late-venous) were considered late-phase, with a similar or higher venous contrast than arterial contrast.

### 2.3. Reference Standard and Annotation

Ground-truth occlusions were annotated on the occlusion site on CTA. Occlusion-negative cases did not contain annotations. Annotations in the detector training and external test cohorts were labeled by two neuroradiologists with six years of experience and reviewed by a senior neuroradiologist with ten years of experience, using the software ITK-SNAP (http://www.itksnap.org/). Medium vessel occlusions were annotated with a sphere of 15-voxel diameter and large vessel occlusions were labeled with a 30-voxel diameter, centered at the most proximal point of loss of contrast in the axial direction [10]. Annotations in the threshold-development subset were provided as thrombus labels derived from the causing occlusions by two neuroradiologists with 10 and 15 years of experience, respectively [22].

### 2.4. Baseline Vessel Occlusion Detector

The baseline occlusion detector was trained with nnDetection [23] using a RetinaNet architecture [24], following the configuration of Brugnara et al. [10]. The detector outputs 3D bounding boxes with associated confidence scores in the range 0.0–1.0. The baseline detector was trained on 900 early-phase CTA volumes, using a validation set of 22 late-phase cases to determine a confidence threshold that removes low-confidence detections while prioritizing sensitivity, optimizing the F2-score at an occlusion level. The selected confidence threshold was 0.37 and was fixed for all subsequent experiments.

### 2.5. Time–Vessel Maps from CT Perfusion

To extract anatomical and temporal information from CTP as time-vessel maps, CTP frames were first registered to CTA using a multi-resolution Euler transform implemented in SimpleElastix [25,26,27,28]. Non-informative frames acquired before contrast arrival or during secondary cardiac cycles were excluded using time-attenuation curves extracted from internal carotid arteries. Internal carotids were segmented using a specialized algorithm for tubular structure segmentation in nnU-Net [29]. The remaining frames were smoothed with a moving-average filter in scikit-image [30]. Intracranial locations were derived via skull-stripping using TotalSegmentator [31]. The CTP curation process is summarized in Figure 2a.

From the curated CTP scans, intracranial vessel locations were identified from a variation image, computed as the difference between the 90th and 10th image percentiles across frames. Regions of high temporal variation were separated from background and parenchyma using k-means clustering with k = 3 in scikit-learn [32]. Temporal information was obtained from a time image encoding the timestep of maximum intensity. Contrast arrival time values were normalized from 0 to 1 to represent relative vascular filling order, with early-filling vessels represented with low values and late-filling vessels with higher values. The final time-vessel map was obtained by masking the time image with the intracranial locations obtained via k-means clustering. The time-vessel map extraction process from curated CTP scans is depicted in Figure 2b.

### 2.6. Constraint-Based Post-Processing Correction Framework

Based on the derived time–vessel maps, three complementary constraints were defined to suppress incorrect occlusion detections and were applied to the detector outputs:

**R1** **(anatomical constraint).** *Detections beyond a distance threshold from vessel truncation points were removed. Truncation points were derived from skeletonized vessels of the time–vessel map and represent potentially occluded vessels.*

**R2** **(temporal constraint).** *Detections in vessels whose contrast-arrival time exceeded a threshold were removed, targeting late-filling vessels that may be enhanced in late-phase CTA and are inconsistent with arterial occlusions.*

**R3** **(spatial prior).** *Detections outside a fixed spatial region in the posterior and superior brain were removed. The allowed region was defined using the distribution of annotated occlusions relative to the brain centroid, including a relative margin between the brain centroid and brain limits in posterior and superior directions. This margin was frozen before external evaluation.*

A summary of the post-processing constraints is presented in Figure 2d.

### 2.7. Parameter Selection, Blinding, and Sensitivity Analysis

The baseline detector was applied to the threshold-development subset, and the three constraint parameters (the distance threshold for R1, the temporal threshold for R2, and the spatial margin for R3) were selected on that subset. The selection process aimed to reduce the number of incorrect occlusions while strictly preserving occlusion-level sensitivity. Parameter combinations that reduced occlusion-level sensitivity were rejected, since preserving true occlusions is a critical requirement in AIS. All parameters were frozen before application to the external test cohort and were not subsequently altered. An ablation experiment on the threshold-development subset assessed all constraint combinations (Section 3.5).

The selected parameters included a distance threshold of 12 voxels (≈6 mm at an approximate median in-plane of 0.5 mm), a relative contrast-arrival-time threshold of 0.40, and a 30% margin in the superior and posterior brain regions with respect to the brain centroid. Because these thresholds were fixed on 32 cases, we additionally assessed their stability through a systematic parameter sweep in Supplementary Table S1.

### 2.8. CTA-to-Time–Vessel Map Generator

To enable application in patients without CTP, a residual U-Net implemented in nnU-Net 29] was trained to estimate time–vessel maps directly from CTA. Input volumes were skull-stripped, with regions outside the brain set to −1024 HU. In this way, the generator could exclusively focus on intracranial locations. The network had three output heads estimating distance to intracranial vessels, vessel segmentation, and contrast-arrival time. The distance and time heads were trained with Mean Absolute Error losses against vessel-distance maps and ground-truth time–vessel maps, respectively, and the segmentation head used a loss function designed for tubular structures. An additional edge-enhancement loss sharpened vessel edges [33]. The distance and segmentation outputs defined the intracranial locations of the estimated time-vessel map, and the time output provided the temporal values. A graphical description of the generator is included in Figure 2c.

The generator was trained on the 223 scans from the generator training cohort and evaluated on the 32 scans from the threshold-development subset. The threshold-development subset was also used to evaluate the generator so that generator-estimated and CTP-derived maps were available for the same cohort, enabling measurement of the influence of generator-estimated maps on the post-processing framework. Generator fidelity was quantified using the Dice coefficient for vessel segmentation and Mean Absolute Error for vessel-distance and contrast-arrival-time estimation.

**Figure 4:**
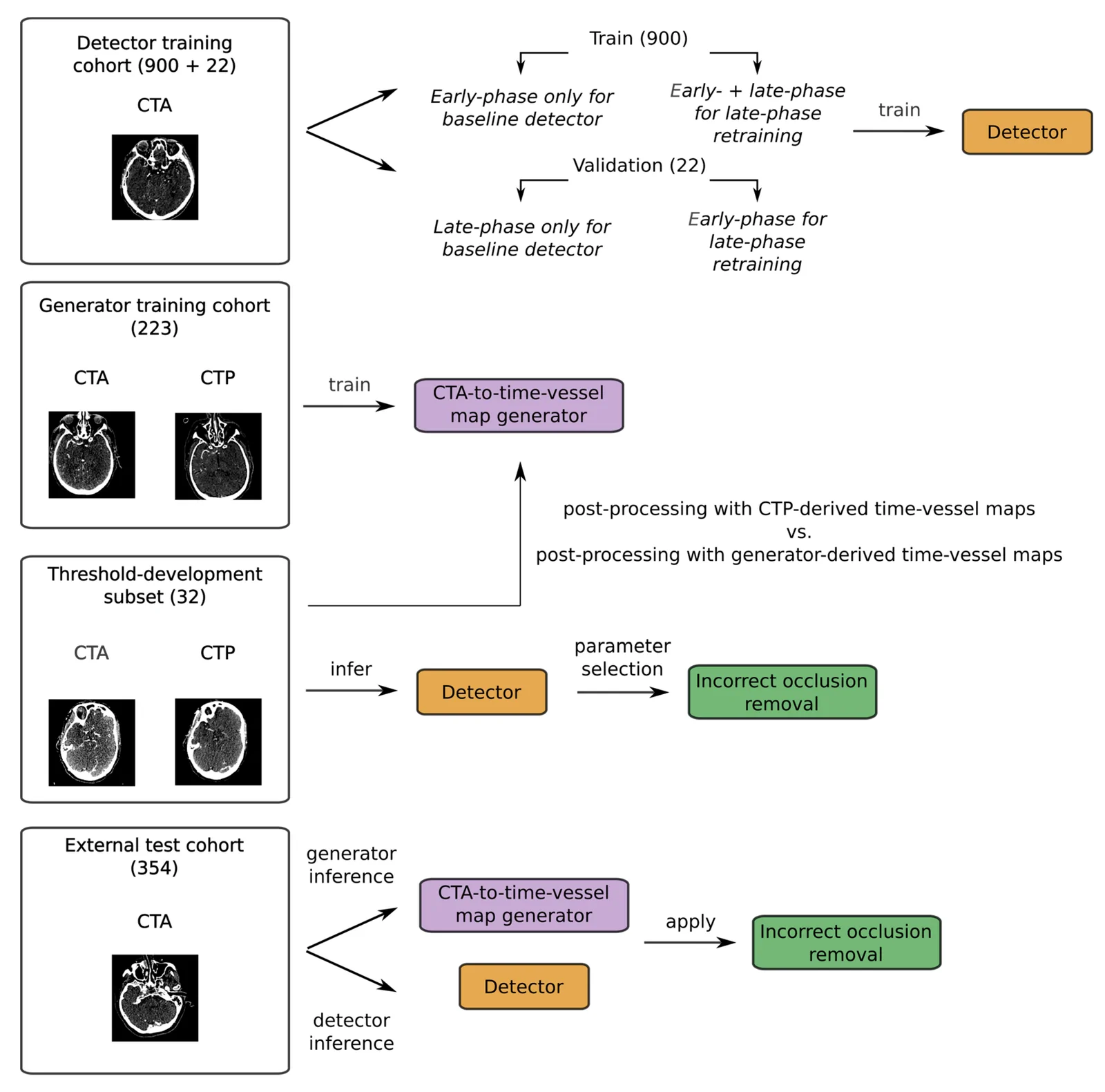
Interaction diagram between cohorts and elements used in the experimental setup. Different colors depict different pipeline elements.

Figure 4 represents the different cohorts and elements in the experimental setup (detectors, CTA-to-time–vessel map generator, incorrect occlusion removal post-processing pipeline).

### 2.9. Comparison Approaches

To assess the direct use of late-phase data during detector training, an additional detector was trained from scratch including the 22 late-phase scans from the detector training cohort, using the same configuration as the baseline detector. This configuration assessed the inclusion of scarce late-phase data during training, termed “late-phase retraining”. Since only 22 late-phase cases were available, this comparison is data-limited and does not represent large-scale late-phase training.

To assess whether temporal and anatomical vascular information is better incorporated during training, an extra-channel detector was trained with the estimated time-vessel map supplied as an additional input channel. Apart from this additional channel, the remaining architecture matched the architecture of the baseline detector. The approach is termed “time-vessel map (extra channel)”.

For fair comparison with the baseline detector, the additional detectors presented here (“late-phase retraining” and “time-vessel map extra channel”) were assigned the same low-confidence threshold as the baseline detector (0.37), so that performance differences reflect the detector and post-processing mechanisms rather than differences in threshold selection.

### 2.10. Metrics and Statistical Analysis

Performance was evaluated at both the occlusion and patient levels after applying the confidence threshold determined on the detector training cohort. The occlusion-level metrics were sensitivity, the number of incorrect detections per scan, and the area under the Free-Response Operating Characteristic curve (FROC), which captures the trade-off between sensitivity and incorrect detections [34]. Patient-level scores were obtained as the maximum confidence across all detections in a scan. Patient-level metrics were the area under the Receiver Operating Characteristic curve (AUROC), the area under the precision–recall curve (AUPRC), and specificity measured at 95% sensitivity. The 95% sensitivity operating point was chosen to evaluate performance at a high fixed sensitivity, since the post-processing framework is intended for triage, where missed occlusions carry high clinical cost [35]. FROC, AUROC, AUPRC, and specificity at 95% sensitivity were expressed as percentages, instead of ratios. Since all patients in the threshold-development subset were occlusion-positive, patient-level metrics were not computed for the threshold-development subset.

We obtained 95% confidence intervals via 1000-iteration bootstrap resampling with replacement at the patient level. Paired bootstrap tests compared the evaluated detectors on the external test cohort, recording metric differences on the same resampled patients (Δ). Two-sided *p*-values below 0.05 were considered significant.

## 3. Results

### 3.1. Patient Population

The final study population, after applying all exclusion criteria, comprised 922 patients in the detector training cohort (72 ± 15 years, 53% female), 32 patients in the threshold-development subset (64 ± 13 years, 34% female), 223 patients in the generator training cohort (71 ± 13 years, 51% female), and 354 patients in the external test cohort (75 ± 8 years, 52% female). Cohort characteristics are shown in Table 1, and additional imaging parameters are included in Supplementary Table S2.

### 3.2. Constraint-Based Post-Processing Reduces Incorrect Occlusion Detections in Late-Phase CTA Scans from External Centers

To determine the effect of post-processing correction, the baseline detector was applied to the external test cohort scans, with and without post-processing. Results for both techniques are shown in the first two rows of Table 2. Post-processing correction reduced the incorrect occlusion detections per scan from 3.9 to 2.1 (Δ = −1.8, 95% CI: −2.0 to −1.6, *p* < 0.0001), while keeping occlusion-level sensitivity unchanged at 80 (Δ = 0.0, 95% CI: 0.0 to 0.0, *p* = 1). FROC improved from 71 to 77 (Δ = 5, 95% CI: 3 to 8, *p* < 0.0001). At the patient level, AUROC increased from 83 to 88 (Δ = 4, 95% CI: 0 to 7, *p* = 0.06), specificity at 95% sensitivity improved from 21 to 40 (Δ = 13, 95% CI: −12 to 24, *p* = 0.24), and AUPRC increased from 61 to 72 (Δ = 9, 95% CI: 1 to 17, *p* = 0.03). Despite the improvements at the patient level, only AUPRC reached significance, as only 51 cases in the external test cohort were occlusion-positive. Taken together, constraint-based post-processing reduces incorrect occlusion detections in late-phase scans from external centers, with significant changes at the occlusion level but not at the patient level.

To determine the computational cost of constraint-based post-processing, time was recorded for three steps: skull-stripping using TotalSegmentator 2.18.0 [31], time-vessel map estimation with the CTA-to-time-vessel-generator, and the application of the post-processing constraints (Table 3). The CTA-to-time-vessel map generator accounted for most of the computational effort, as it requires 3D convolutional neural network processing. At the same time, post-processing constraints also occupied a substantial fraction of the time, since R1 required vessel skeletonization of the time-vessel map to obtain vessel truncation points.

**Figure 5:**
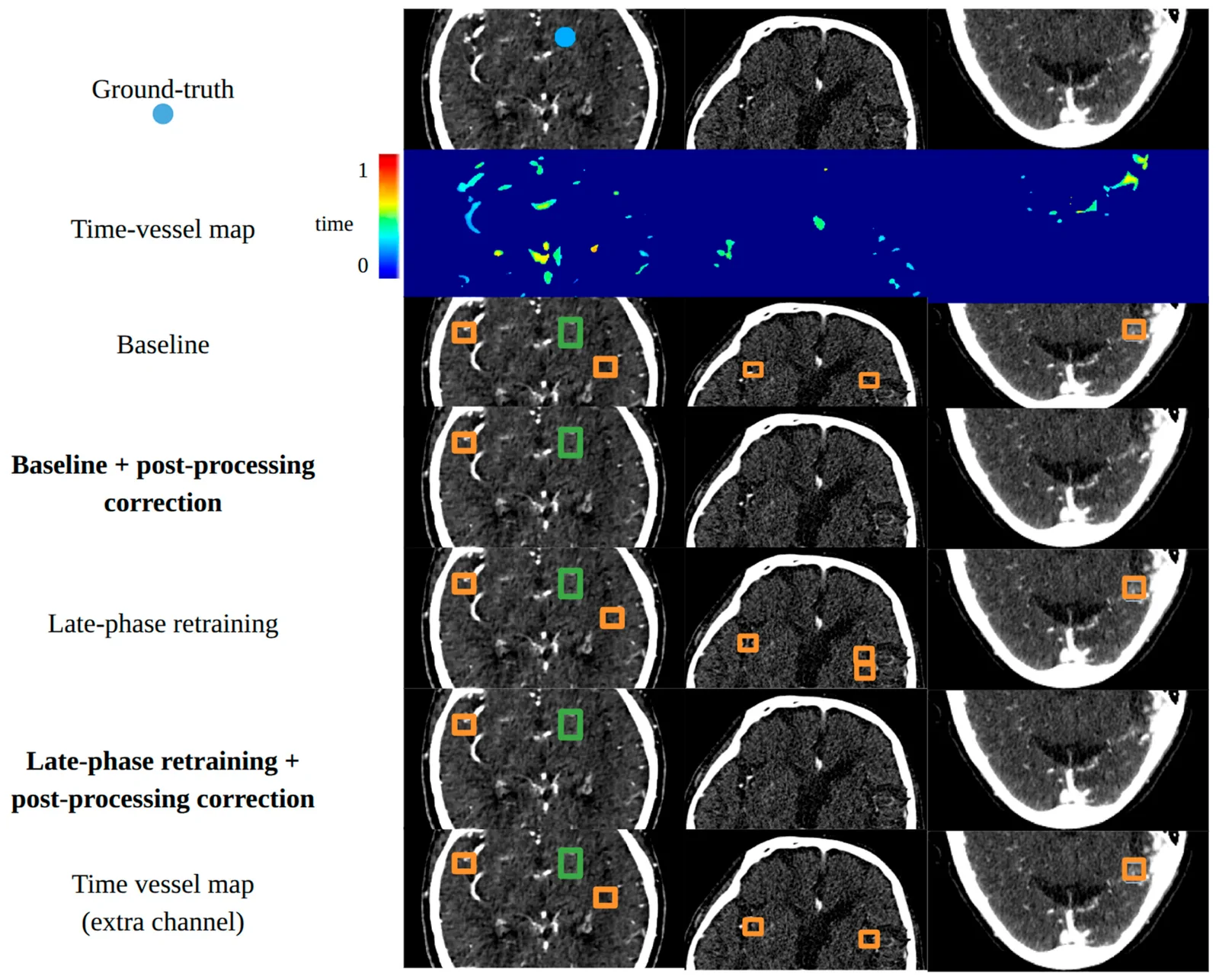
Qualitative results on the external test cohort, for the benchmarked methods, including estimated time-vessel maps with the CTA-to-time-vessel map generator. Blue blobs denote occlusion annotations; orange boxes denote incorrect occlusion detections; green boxes denote true occlusion detections. Methods in bold use the post-processing correction framework.

A qualitative analysis is shown in Figure 5, with occlusion detections for three cases across all tested methods. Low-confidence boxes were removed using the confidence threshold from the detector training cohort’s validation set. The post-processing targets detections in non-occluded vessels in the first two columns and in late-filling posterior vessels in the last row.

The external test cohort comprised four different centers, including related results in Supplementary Tables S3–S6.

### 3.3. Constraint-Based Post-Processing Is Also Helpful When the Detector Has Observed Late-Phase Cases During Training, Although to a Lower Extent

To determine the behavior of post-processing correction with detectors that have already seen late-phase cases during training, post-processing correction was also applied on top of a detector trained using the late-phase scans available in the detector training cohort (“late-phase retraining”). Results are shown in the third and fourth rows of Table 2. The incorrect occlusions per scan were reduced from 2.8 to 1.9 (Δ = −0.9, 95% CI: −1.1 to −0.8, *p* < 0.0001), keeping occlusion-level sensitivity unchanged at 80 (Δ = 0, 95% CI: 0 to 0, *p* = 1), while FROC did not change, remaining at 77 (Δ = 0, 95% CI: 0 to 2, *p* = 0.09). At a patient level, the changes were smaller: AUROC increased from 89 to 90 (Δ = 2, 95% CI: −1 to 4, *p* = 0.14), specificity at 95% sensitivity improved from 40 to 51 (Δ = 14, 95% CI: 4 to 19, *p* = 0.03), and AUPRC increased from 77 to 78 (Δ = 1, 95% CI: −2 to 4, *p* = 0.49). Consequently, post-processing correction also removes incorrect occlusions from detectors already trained on late-phase scans, although the improvements are more slight, since part of the late-phase cues leveraged by the post-processing correction module in the baseline detector were already learned during training on late-phase scans.

### 3.4. Attaching Time-Vessel Maps During Detector Training Also Leads to Improvements, at the Cost of Lower Occlusion Sensitivity

To examine the effect of time-vessel maps during training, an additional detector termed “time-vessel map extra channel” included the time-vessel maps as extra input channels. Compared with post-processing correction, incorrect occlusions per scan reduced further from 2.1 to 1.4 (Δ = −0.7, 95% CI: −0.8 to −0.6, *p* < 0.0001), while FROC reduced from 77 to 75 (Δ = −2, 95% CI: −6 to 3, *p* = 0.42) and occlusion-level sensitivity reduced from 80 to 78 (Δ = −1, 95% CI: −2 to 0, *p* = 0.004). At the patient level, incorporating time-vessel maps during training slightly improved performance: AUROC from 88 to 91 (Δ = 3, 95% CI: −1 to 7, *p* = 0.15), specificity at 95% sensitivity from 40 to 47 (Δ = 7, 95% CI: −11 to 12, *p* = 0.74), and AUPRC from 72 to 84 (Δ = 12, 95% CI: 3 to 22, *p* = 0.006). Despite the patient-level improvements, the drop in occlusion sensitivity implied the loss of true occlusions estimated by the baseline model. Taken together, incorporating time-vessel maps during training can yield patient-level improvements but also a slight drop in occlusion-level sensitivity, which is risky for AIS diagnosis.

### 3.5. Temporal Information Is Most Relevant for Constraint-Based Post-Processing

To analyze the contribution of each constraint, the post-processing correction pipeline was applied to the threshold-development subset with different constraints activated or deactivated (Table 4). The combination of all constraints gave the best overall performance, reducing incorrect occlusions per scan from 2.2 (95% CI: 2.1–2.6) to 1.7 (95% CI: 1.4–1.8), keeping a constant occlusion sensitivity with respect to the baseline detector of 59 (95% CI: 48–65) and reaching the highest FROC of 49 (95% CI: 35–63). Among individual constraints, the temporal constraint R2 provided the largest individual improvements, closely matching the full set, though with slightly lower FROC. The anatomical (R1) and spatial-prior (R3) constraints also reduced incorrect occlusions per scan, though less than R2, and contributed to the FROC gain of the full set. These results indicate that the temporal constraint, targeting detections in late-filling vessels, provides the greatest improvement, while the anatomical constraint and spatial prior provide complementary improvements.

Performance in the threshold-development cohort for other constraint threshold values in a parameter sweep is included in Supplementary Table S1.

### 3.6. CTA-to-Time-Vessel Map Generator Presents an Acceptable Performance That Can Be Improved

**Figure 6:**
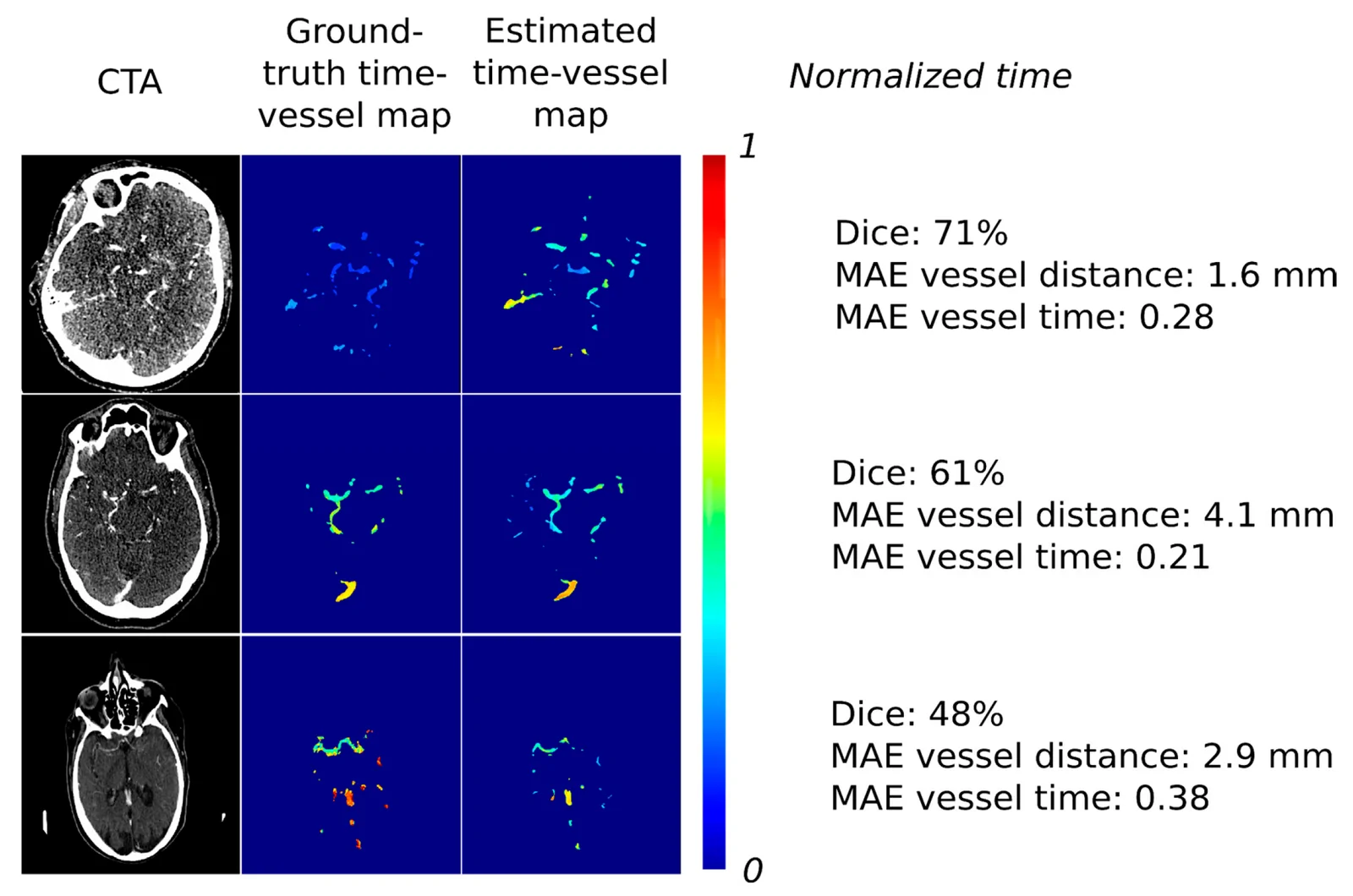
Best, median, and worst cases (with metrics) from the threshold-development subset. CTA, CT angiography; MAE, mean absolute error.

To study the fidelity of the CTA-to-time-vessel map generator, the generated time-vessel maps in the threshold-development subset were compared with the CTP-derived maps. For vessel segmentation, a Dice coefficient of 60 (95% CI: 58–62) was obtained. For vessel distance estimation and time estimation, Mean Absolute Errors of 2.3 (95% CI: 2.1–2.5) mm and 0.29 (95% CI: 0.27–0.31) were obtained, respectively. These values are also included in Supplementary Table S7. Overall, the generator shows acceptable but improvable performance: the vessel-distance error is comparable to the size of some intracranial vessels, and the time-estimation error occupies a meaningful fraction of the 0.0–1.0 normalized time scale. Representative best, median, and worst cases in the threshold-development subset are shown in Figure 6.

### 3.7. CTA-to-Time-Vessel Map Generator Influences the Performance of Constraint-Based Post-Processing

**Figure 7:**
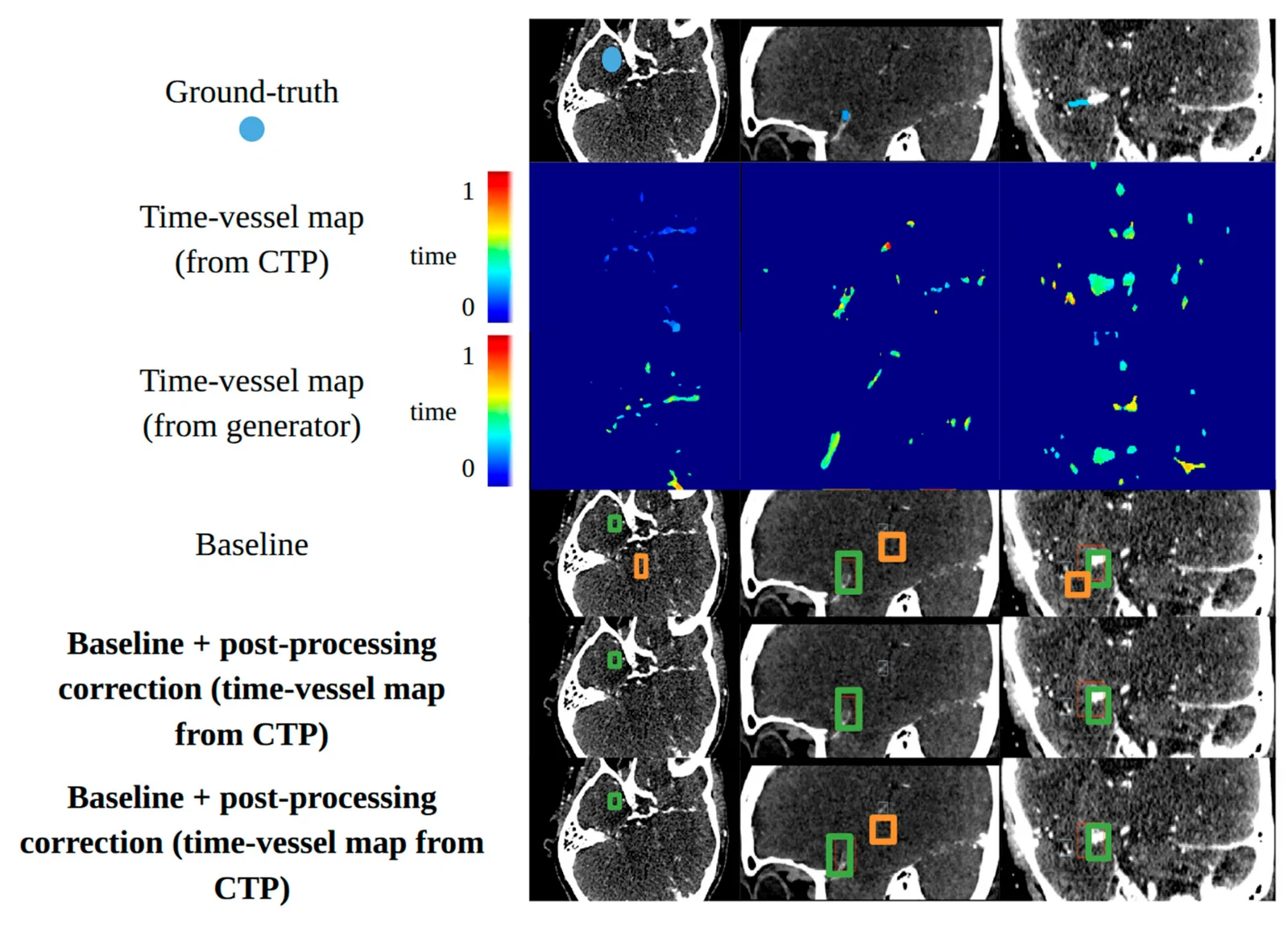
Influence of the CTA-to-time-vessel map generator in the threshold-development subset, across three cases, including CTP-derived vessel maps, generator-derived time-vessel maps, and performance for the baseline detector, with and without post-processing correction using both time-vessel map types. Blue blobs denote ground-truth occlusions; orange boxes denote incorrect occlusion detections; green boxes denote true occlusion detections. Methods in bold use the post-processing correction framework.

To determine the generator’s influence on downstream post-processing correction, the threshold-development subset served as the generator’s evaluation set, providing both CTP-derived time-vessel maps and generated time-vessel maps for the same set of cases. Results for the baseline detector and for post-processing with both time-vessel map types are included in Table 5. Post-processing with generated maps also reduced incorrect occlusion detections per scan to 1.8 (95% CI: 1.7–2.0), though to a lower extent than CTP-derived time-vessel maps (1.7). FROC improved to 48 (95% CI: 35–62), slightly below the CTP-derived time-vessel maps (49). Occlusion sensitivity degraded slightly, from 59 (95% CI: 48–65) to 56 (95% CI: 50–62), due to the removal of a true occlusion in a carotid location underrepresented in the annotations. Taken together, generated time–vessel maps also have the potential to remove incorrect occlusion detections, although occlusion-level sensitivity degrades slightly. Qualitatively, we can observe in Figure 7 that post-processing with generator-derived maps removed incorrect occlusion detections in the cases from the first and last column but not from the second column.

## 4. Discussion

### 4.1. Overview

Late-phase CTA remains challenging for computer-aided vessel-occlusion detection in AIS because delayed venous enhancement can mimic arterial occlusions, leading to incorrect occlusion detections. This is clinically relevant, as late acquisitions occur in practice, particularly in non-specialized centers [13]. We present a constraint-based post-processing correction framework that integrates vascular anatomy and contrast-arrival time from CTP to suppress incorrect occlusion detections in late-phase CTA. In an independent, multicenter external test cohort, the framework reduced incorrect occlusion detections per scan from 3.9 to 2.1 for a baseline detector and from 2.8 to 1.9 for a detector already exposed to late-phase cases during training. In both settings, occlusion sensitivity was unchanged in the external test cohort, showing that the post-processing correction did not remove true occlusions.

### 4.2. Comparison to Methods Using Temporal Information for Vessel Occlusion Detection

Several automated approaches incorporate time-resolved information for large-vessel occlusion detection. A two-stage network for large-vessel occlusion classification and localization on multiphase CTA achieved 83% sensitivity, 90% specificity, and an AUROC of 89 [19]. A symmetry-based analysis of dynamic CTA derived from CTP reported strong performance on easy cases but marked degradation on high-difficulty occlusions (sensitivity 53%, specificity 11%), with perfusion data improving specificity [20]. Another method used intermediate temporal representations, where a normalized time-to-signal map fed to a convolutional neural network detected anterior-circulation occlusions in multiphase CTA with 95% sensitivity and 92% specificity (AUROC 98) [21]. Nevertheless, the objective of these methods differs from those of our study. While previous methods aimed to improve detection of large-vessel occlusions by leveraging temporal information from 4D modalities, we aimed to improve detection robustness in late-phase CTA by leveraging CTP-derived information acquired together with single-phase CTA, focusing also on medium-vessel occlusions. Our extra-channel detector was an end-to-end detector that exploited temporal information during detector development, in a similar spirit to [19,20,21]. In this case, the extra-channel detector improved AUROC and AUPRC but reduced occlusion sensitivity (Table 2), while constraint-based post-processing preserved sensitivity. Consequently, when sensitivity is the priority, the proposed post-processing correction is preferable to training-time integration of temporal information.

### 4.3. Implications of Constraint-Based Post-Processing in Late-Phase Scans from External Centers

The primary contribution of the proposed constraint-based post-processing is a reduction in incorrect occlusion detections and unchanged sensitivity across four external centers, even for detectors that have already been exposed to a small quantity of late-phase cases during training. This matters clinically because incorrect occlusion alerts on late-phase CTA may trigger unnecessary downstream review and erode trust in automated triage. A method that reduces incorrect alerts without degrading sensitivity can decrease alert fatigue while keeping the miss rate for true occlusions unchanged. Conversely, any drop in sensitivity directly affects missed occlusions, which are the dominant clinical risk in AIS detection. We therefore prioritized sensitivity preservation. The proposed pipeline takes an average of almost four minutes for post-processing. Most effort is spent on estimating the time-vessel map and skeletonizing vessels in the time-vessel map for the application of R1. These times can be optimized to facilitate deployment for real clinical triage. Future work will focus on optimization through parallelization and lighter components where possible.

The suppression rules of the proposed post-processing framework, although fixed on only 32 cases, remained effective in the external test cohort, suggesting that the targeted incorrect-detection patterns are at least partially consistent across datasets. Improvements for detectors that had already seen late-phase cases during training were more limited, probably because these detectors already partly handle these shifted cases. Late-phase cases are infrequently acquired [13], making it difficult to have balanced cohorts, in terms of phase, to train detectors. Notably, the proposed post-processing also improved performance for a detector already exposed to late-phase cases during training, indicating that post-processing may remain beneficial even when some late-phase data are available for training. Supplying time-vessel maps during training also improved results over the baseline detector, yielding the lowest incorrect occlusion detections per scan and the highest AUROC and AUPRC. However, this approach also reduced occlusion sensitivity, removing true occlusions. This method was characterized by an increased aggressiveness toward incorrect occlusion removal compared to post-processing, sometimes at the cost of missing true occlusions. Given the design choice to prioritize occlusion sensitivity for AIS detection, post-processing was preferable. The fact that only 51 cases in the external test cohort were occlusion-positive led to wide CIs and non-significant differences in AUROC, AUPRC, and specificity at 95% sensitivity. Consequently, the patient-level results should be read as suggestive rather than confirmatory.

### 4.4. Individual Constraint Contributions

The activation and deactivation of the post-processing constraints in the threshold-development subset showed that temporal constraint R2 led to the largest reduction in incorrect occlusion detections per scan, preserving occlusion sensitivity. This is consistent with delayed vessel filling on late-phase scans, which mimics arterial occlusions and can produce incorrect occlusion detections. R2 helped identify these vessels and remove detections within them. The anatomical constraint R1 and the spatial prior R3 contributed complementarily, jointly yielding the highest FROC when all constraints were applied together, by targeting detections in non-occluded vessels and outside intracranial regions of high occlusion prevalence. Threshold choice affected performance (Supplementary Table S1), since small changes in the constraint thresholds slightly altered results, most notably the margin for the spatial prior R3, where restricting the allowed region to only 20% of the superior and posterior brain regions relative to the brain centroid decreased occlusion sensitivity. At the same time, increases in the normalized time threshold also led to increases in the incorrect occlusion detections per scan, since not so many detections in late-filling vessels were targeted.

The additional detectors (“late-phase retraining” and “time-vessel map (extra channel)”) were also evaluated in the threshold-development subset (Supplementary Table S8 and Supplementary Figure S1). Post-processing correction improved results for the late-phase retraining detector, although with a slight decrease in occlusion sensitivity in this specific subset, whereas supplying time-vessel maps during detector training improved FROC and occlusion sensitivity, but increased the number of incorrect occlusions per scan.

### 4.5. Implications of the CTA-to-Time-Vessel Map Generator

The CTA-to-time-vessel map generator was designed to estimate time-vessel maps for cases lacking CTP. Quantitatively, the generator’s performance in the threshold-development subset was acceptable but could be improved. A Dice coefficient of 60 implies a 60% overlap with the vessels of the CTP-derived time-vessel maps. The vessel-distance error of 2.3 mm is comparable to the size of some intracranial vessels [36], while the temporal estimation error of 0.29 corresponds to a non-trivial fraction of the normalized time range (0.0–1.0). Compared with CTP-derived maps, most vessels appeared to be recovered (Figure 6 and Figure 7), with greater difficulty on small vessels and vessel edges. The estimated vessel edges were comparatively smooth, which may have contributed to a lower Dice score. Temporal values were recovered with some accuracy, although the generator struggled with very early- or very late-filling vessels, which were probably less common in the generator training data, tending to assign intermediate time values to these vessels. A representative failure mode is shown in the last case of Figure 6, where low vessel contrast in the input CTA prevented recovery of all intracranial vessels of interest. We also note that CTP-derived time-vessel maps may present distortions due to variability in contrast-bolus timing, extensive collateral circulation, or atypical vascular anatomy. Hence, the comparison of the generated maps to CTP-derived maps should be regarded as indicative rather than definitive.

Deviations in the generated maps led to the slightly lower performance of the post-processing framework than CTP-derived maps, with a small decrease in occlusion sensitivity (Table 5). Despite these limitations, the post-processing correction framework with generator-derived time-vessel maps reduced incorrect occlusion detections per scan while preserving occlusion sensitivity in the external test cohort. The generator therefore improved performance in late-phase cases without CTP. However, directly leveraging CTP-derived maps, if CTP is available, is likely to yield the best performance.

### 4.6. Limitations and Future Work

Several limitations should be acknowledged. First, the method was evaluated only on retrospective late-phase data. The method also requires phase classification of the CTA, where small errors in arterial or venous contrast measurement could cause misclassification in ambiguous cases. No reference-independent methods to determine phase without using arterial and venous contrast values were found. Hence, phase classification errors could not be quantitatively determined. Prospective studies should assess the impact on reader decision-making and on early-phase cases, which was beyond the scope of this proof-of-concept study.

Second, threshold selection was performed on a relatively small subset of late-phase scans from younger patients with a higher proportion of males than the other cohorts (Table 1). However, we observed a positive effect of the selected thresholds in the external test cohort (Table 2), which contained more than ten times the number of cases in the threshold-development subset (*N* = 354). All patients in the threshold-development subset were occlusion-positive, since they originated from the CLEOPATRA dataset collection, which does not include healthy controls [16,22]. Patients with late-phase CTA, thin-slice CTP matching the CTA spacing, and occlusion annotations were difficult to obtain, resulting in a restricted dataset that could lead to overfitted constraint thresholds. The use of scalar thresholds may also oversimplify the problem of vessel occlusion detection in phase-shifted CTA. Future work should enlarge the threshold-development subset, potentially by annotating additional cases with late-phase CTA and thin-slice CTP, and explore more elaborate constraints that increase robustness to phase shifts.

Third, only 22 late-phase scans were available in the detector training cohort to study the inclusion of late-phase information during training, as this cohort was acquired predominantly in the early phase [10]. A larger number of late-phase cases may further improve detector robustness to phase shifts, potentially reducing the need for post-processing, which should be investigated in future work. However, the proposed method is still beneficial when late-phase scans are scarce.

Finally, the proposed framework relied on a CTA-to-time-vessel map generator for cases without CTP. The generator helped to improve performance in an external cohort without CTP while keeping sensitivity unchanged but struggled with vessel edge retrieval and precise time estimation for very early and very late filling vessels. This may impact R2, since substantial errors in time estimation may affect performance in vessels with a relative timing close to R2’s threshold, as boxes in these vessels could be potentially removed by mistake. The use of the CTA-to-time-vessel map generator in the threshold-development cohort led to a slight decrease in occlusion sensitivity. Consequently, alternative generation architectures beyond U-Net should be explored in future studies [37,38].

## 5. Conclusions

We present a constraint-based post-processing correction framework that reduces incorrect vessel-occlusion detections in late-phase CTA from four external centers while preserving occlusion-level sensitivity. This pattern was observed in a detector exclusively trained on early-phase scans and in a detector already exposed to late-phase scans during training. However, the improvements were smaller in the second case. By incorporating vascular anatomy and contrast-arrival time derived from CTP, the framework improves the robustness of computer-aided detection in late-phase acquisitions, where venous enhancement can mimic arterial occlusions. The results further suggested that sensitivity-preserving post-processing may be preferred to integrating the same information during detector training, since anatomy and temporal vascular information inclusion during training led to a decrease in occlusion sensitivity. From the different post-processing constraints, temporal constraints contributed the most to overall improvements. We additionally developed a generator capable of estimating the required anatomical and temporal information for post-processing in cohorts lacking CTP. Using this generator on a subset with CTP scans also improved performance, although not to the same level as handling native CTP scans, since the generator managed to estimate intracranial vessels but was improvable for vessel edge and contrast arrival time estimation. As the next steps, this proof-of-concept approach requires prospective application and time optimization to continue analyzing benefits in clinical workflows, aiming to reduce incorrect alerts that erode trust in automated AIS detection systems.

## Figures and Tables

**Table 1:** Demographic and imaging characteristics by cohort. Ages reported as mean (standard deviation). CTA, CT angiography; CTP, CT perfusion; *N*, number of patients; NA, non-applicable.

Characteristic	Detector Training	Threshold-Development Subset	Generator Training	External Test
*N*	922	32	223	354
Occlusion-positive patients	543	32	223	51
No. of occlusions	581	32	223	51
Sex (% female)	53	34	51	52
Age (years)	72 (15)	64 (13)	71 (13)	75 (8)
Manufacturer	Siemens (100%)	Siemens (100%)	Siemens (100%)	Siemens (42%)/Philips (58%)
CTA spacing (mm)	0.49/0.49/0.51	0.43/0.43/0.41	0.46/0.46/0.53	0.54/0.54/0.66
CTP spacing (mm, s)	NA	0.42/0.42/0.79/1.56	0.42/0.42/0.87/1.57	NA

**Table 2:** Vessel-occlusion detection performance in the external test cohort (*N* = 354). Results are shown for the baseline detector, the baseline detector with post-processing correction, a detector including late-phase scans in the training set (with and without post-processing correction), and a detector using the time–vessel map as an additional input channel. Each cell reports the mean with the 95% confidence interval in brackets, from 1000-iteration bootstrap resampling with replacement at the patient level; specificity is reported at a fixed 95% sensitivity operating point. The best value per column is in bold; note that the time–vessel map (extra channel) detector attains the highest AUROC and AUPRC but does not preserve occlusion-level sensitivity (Section 3.4). FROC, Free-Response Operating Characteristic curve; AUROC, area under the Receiver Operating Characteristic curve; AUPRC, area under the precision–recall curve.

Method	Incorrect Occlusions/Scan	FROC	Occlusion Sensitivity	AUROC	Specificity at 95% Sensitivity	AUPRC
Baseline	3.9 (3.6–4.2)	71 (60–81)	80 (69–91)	83 (75–91)	21 (9–47)	61 (46–74)
Baseline + post-processing correction	2.1 (1.9–2.3)	77 (66–86)	80 (69–91)	88 (82–94)	40 (18–61)	72 (62–84)
Late-phase retraining	2.8 (2.7–3.0)	77 (65–87)	80 (69–91)	89 (83–95)	40 (20–53)	77 (67–86)
Late-phase retraining + post-processing correction	1.9 (1.8–2.1)	**77 (66–87)**	80 (69–91)	90 (85–95)	**51 (36–68)**	78 (70–86)
Time–vessel map (extra channel)	**1.4 (1.3–1.6)**	75 (63–85)	78 (67–89)	**91 (84–98)**	47 (29–97)	**84 (74–93)**

**Table 3:** Mean per-scan processing time for each component of the pipeline. Times were measured on a workstation with an NVIDIA GeForce RTX 4090 GPU (24 GB VRAM) and an AMD Ryzen 9 7950X CPU (16 cores, 32 threads). Brain segmentation and the CTA–to–time–vessel map generator ran on the GPU, and the post-processing ran on the CPU. Values are reported as the mean (95% CI) across the 354 cases in the external test cohort.

	Brain Segmentation	CTA-to-Time-Vessel Map Generator	Post-Processing Constraints	Total
Time (seconds)	34.4 (34.3–34.6)	144.2 (139.4–149.0)	56.5 (54.6–58.4)	236.3 (230.9–241.7)

**Table 4:** Vessel-occlusion detection performance in the threshold-development subset under different constraint combinations, including baseline performance (*N* = 32). The 95% CI is in brackets, from 1000-iteration bootstrapping with replacement at the patient level. Best value per column in bold. FROC, Free-Response Operating Characteristic curve.

Method (*N* = 32)	FROC	Incorrect Occlusions/Scan	Occlusion Sensitivity
Baseline	46 (33–60)	2.2 (2.1–2.6)	59 (48–65)
Baseline + R1	46 (33–61)	1.9 (1.8–2.2)	59 (48–65)
Baseline + R2	48 (35–63)	**1.7 (1.4–1.8)**	59 (48–65)
Baseline + R3	46 (33–61)	1.9 (1.8–2.2)	59 (48–65)
Baseline + R1 + R2	48 (35–61)	1.9 (1.5–2.1)	59 (48–65)
Baseline + R1 + R3	46 (33–61)	1.9 (1.8–2.2)	59 (48–65)
Baseline + R2 + R3	48 (35–63)	**1.7 (1.4–1.8)**	59 (48–65)
**Baseline + R1 + R2 + R3**	**49 (35–63)**	**1.7 (1.4–1.8)**	59 (48–65)

**Table 5:** Vessel occlusion detection performance in the threshold-development subset for the baseline detector and post-processing correction using CTP-derived time-vessel maps and generator-derived maps. Best value per column in bold. The 95% CI is in brackets, from 1000-iteration bootstrapping with replacement at the patient level.

Method (*N* = 32)	Time-Vessel Map Used	FROC	Incorrect Occlusions/Scan	Occlusion Sensitivity
Baseline	NA	46 (33–60)	2.2 (2.1–2.6)	**59 (48–65)**
Baseline + post-processing correction	CTP-derived	**49 (35–63)**	**1.7 (1.4–1.8)**	**59 (48–65)**
Baseline + post-processing correction	Generator-derived	48 (35–62)	1.8 (1.7–2.0)	56 (50–62)

## Data Availability

The data are not publicly available owing to privacy and ethical restrictions.

## Supplementary Materials

The following supporting information can be downloaded at: https://www.mdpi.com/article/10.3390/diagnostics16182972/s1, S1: Detailed Methods [10,11,22,23,24,25,26,27,28,29,30,31,32,33,39]; S2: Extended Results; Table S1: Parameter sweep for the constraint thresholds on the threshold-development subset (*N* = 32). The distance threshold for R1 (in voxels), the relative contrast-arrival-time threshold for R2, and the spatial margin for R3 (as a percentage of the distance from the brain centroid to the brain limits) were varied jointly. Each cell reports the mean with 95% confidence interval (2.5th–97.5th percentile) from bootstrap resampling; values are integers except the incorrect occlusion detections per scan (one decimal). The Baseline row corresponds to the detector without post-processing. The selected configuration (R1 = 12 voxels, R2 = 0.40, R3 = 30%), which minimized incorrect occlusion detections while preserving occlusion-level sensitivity, is shown in bold. FROC, Free-Response Operating Characteristic curve; Table S2: Imaging data (reconstruction kernel and image dimensions) for the baseline-detector training, generator training, and external test cohorts. Values are reported as mean (standard deviation). For the generator training cohort, italicized statistics correspond to the late-phase threshold-development subset used for parameter selection. CTA, CT angiography; CTP, CT perfusion; Table S3: Stratified results in the external test cohort for late-phase cases in Center 1 (Philips only), with 95% confidence intervals. Best values in bold. FROC, Free-Response Operating Characteristic curve; AUROC, area under the Receiver Operating Characteristic curve; AUPRC, area under the Precision-Recall curve; Table S4: Stratified results in the external test cohort for late-phase cases in Center 2 (Siemens only), with 95% confidence intervals. Best values in bold. FROC, Free-Response Operating Characteristic curve; AUROC, area under the Receiver Operating Characteristic curve; AUPRC, area under the Precision-Recall curve; Table S5: Stratified results in the external test cohort for late-phase cases in Center 3 (Siemens only), with 95% confidence intervals. Only incorrect detections per scan are available, since this subset contained no occlusion-positive cases. Best values in bold. FROC, Free-Response Operating Characteristic curve; AUROC, area under the Receiver Operating Characteristic curve; AUPRC, area under the Precision-Recall curve; Table S6: tratified results in the external test cohort for late-phase cases in Center 4 (Siemens only), with 95% confidence intervals. Best values in bold. FROC, Free-Response Operating Characteristic curve; AUROC, area under the Receiver Operating Characteristic curve; AUPRC, area under the Precision-Recall curve; Table S7. Quantitative results for the CTA-to-time–vessel map generator on the test partition of the generator training cohort (*N* = 51). Values are point estimates with 95% confidence intervals. MAE, mean absolute error; Table S8. Vessel occlusion detection performance on the threshold-development subset (quantitative results); Figure S1. Detection performance on the threshold-development subset (qualitative results), including baseline post-processing with CTP-derived time-vessel maps and baseline post-processing with generator-derived time-vessel maps. Blue blobs denote vessel-occlusion annotations, orange boxes denote incorrect occlusion detections, and green boxes denote true occlusion detections.

## Abbreviations

The following abbreviations are used in this manuscript:

AIS	Acute ischemic stroke
AUPRC	Area under the precision–recall curve
AUROC	Area under the Receiver Operating Characteristic curve
CI	Confidence interval
CTA	Computed tomography angiography
CTP	Computed tomography perfusion
FROC	Free-Response Operating Characteristic curve
MAE	Mean absolute error
nnU-Net	no-new U-Net
