# Supplementary material for: Anatomy- and Time-Based Post-Processing with CT Perfusion-Derived Time–Vessel Maps to Reduce Incorrect Occlusion Detections in Late-Phase CT Angiography
Source: Diagnostics (Basel). 2026 Sep 14;16(18):2972. doi: 10.3390/diagnostics16182972 (PMC13605062; doi:10.3390/diagnostics16182972)
Supplement: Supplementary file 1 [file diagnostics-16-02972-s001.zip › diagnostics-4535093-supplementary.pdf]

Supplementary Materials

Anatomy- and Time-Based Post-Processing with CT Perfusion-Derived Time–Vessel Maps to Reduce Incorrect Occlusion Detections in Late-Phase CT Angiography

Andres Martinez Mora [1,2,3,4,5,6,*], Mahsa Mojtahedi [2,3,7,8], Lucas de Vries [2,3,7,8], Michael Baumgartner [1,9,10], Yannick Kirchhoff [1,10,11,12], Maximilian Zenk [1,5], Katharina Eckstein [1,5,13], Jessica Kachele [1,5,13], Gianluca Brugnara [1,14], Martin Bendszus [15], Alexander Radbruch [14,16], Yvo Roos [2,3], Wim van Zwam [17,18], Robert van Oostenbrugge [17,18], DiederikW. J. Dippel [19], Bart J. Emmer [2,3], Charles Majoie [2], Philipp Vollmuth [1,14], Clara I. Sanchez [3,4], Klaus H. Maier-Hein [1,5,9,10,13,20,21] and Henk A. Marquering [2,3]

1 Department of Medical Image Computing, German Cancer Research Center, 69120 Heidelberg, Germany
2 Department of Radiology and Nuclear Medicine, Amsterdam University Medical Centers, Location AMC, 1105 AZ Amsterdam, The Netherlands
3 Department of Biomedical Engineering and Physics, Amsterdam University Medical Centers, Location AMC, 1105 AZ Amsterdam, The Netherlands
4 Quantitative Healthcare Analysis (QurAI) Group, Informatics Institute, University of Amsterdam, 1098 XH Amsterdam, The Netherlands
5 Medical Faculty Heidelberg, University of Heidelberg, 69120 Heidelberg, Germany
6 Konrad Zuse School of Excellence in Learning and Intelligent Systems (ELIZA), Technical University of Darmstadt, 64289 Darmstadt, Germany
7 Amsterdam Cardiovascular Sciences, 1105 AZ Amsterdam, The Netherlands
8 Amsterdam Neuroscience, 1105 AZ Amsterdam, The Netherlands
9 Helmholtz Imaging, German Cancer Research Center, 69120 Heidelberg, Germany
10 Faculty of Mathematics and Computer Science, University of Heidelberg, 69120 Heidelberg, Germany
11 HIDSS4Health—Helmholtz Information and Data Science School for Health, Karlsruhe Institute of Technology, 76131 Karlsruhe, Germany
12 HIDSS4Health—Helmholtz Information and Data Science School for Health, Karlsruhe Institute of Technology, 69120 Heidelberg, Germany
13 German Cancer Consortium (DKTK), German Cancer Research Center (DKFZ), Core Center Heidelberg, 69120 Heidelberg, Germany
14 Clinic for Neuroradiology, University Hospital Bonn, 53127 Bonn, Germany
15 Department of Neuroradiology, Heidelberg University Hospital, 69120 Heidelberg, Germany
16 Medical Faculty, University of Bonn, 53113 Bonn, Germany
17 Department of Radiology and Nuclear Medicine, Maastricht University Medical Center, 6229 HX Maastricht, The Netherlands
18 School for Cardiovascular Diseases (CARIM), Maastricht University, 6200 MD Maastricht, The Netherlands
19 Department of Neurorology, Erasmus Medical Center, 3015 MD Rotterdam, The Netherlands
20 National Center for Tumor Diseases (NCT), 69120 Heidelberg, Germany
21 Pattern Analysis and Learning Group, Heidelberg University Hospital, 69120 Heidelberg, Germany
* Correspondence: andres.martinezmora@dkfz-heidelberg.de; Tel.: +49-15143319325

S1. Detailed Methods

S1.1. Overview

The proposed pipeline consisted of three components: (1) a baseline vessel-occlusion detector, (2) a post-processing correction framework leveraging vascular anatomical, temporal, and spatial constraints derived from time–vessel maps, and (3) an auxiliary CTA-to-time–vessel map generator for patients without CTP. In addition to these components, CTP data were first preprocessed to extract the time–vessel maps required by the post-processing correction approach. Technical details for each component are provided in the following sections.

S1.2. Patient Population

The baseline-detector training cohort was obtained from Heidelberg University Hospital [1] and comprised patients with vessel-occlusion annotations acquired between March 2010 and February 2020, predominantly in early phases. Scans with insufficient image quality or extracranial occlusions were excluded.

The generator training cohort was derived from CLEOPATRA [2], a series of trials and registries acquired between January 2018 and March 2022, comprising paired CTA and thin-slice CTP scans of AIS patients with slice thickness below 1.5 mm. All patients in this cohort were occlusion positive.

The threshold-development subset included 32 late-phase CTA cases with occlusion annotations drawn from the same data sources as the generator training cohort. This subset was used to fix the parameters of the post-processing framework.

The external test cohort consisted of labelled CTA scans from four external centers acquired between September 2020 and June 2022 [1], without paired CTP data. Unlike the generator training cohort, the external test cohort included both occlusion-positive and occlusion-negative patients. Scans with extracranial occlusions were excluded.

In all cohorts, CTA phase classification was performed using a rule-based classifier comparing Hounsfield-unit enhancement in arterial and venous intracranial regions [3]. Scans were categorized as early-arterial, peak-arterial, equilibrium, peak-venous, or late-venous. Early-arterial and peak-arterial phases were grouped as early phases; the remaining phases were considered late phases.

S1.3. CTP Preprocessing and Time–Vessel Map Derivation

CTP scans were preprocessed before extracting time–vessel maps. Each CTP frame was registered to the corresponding CTA volume using a multi-resolution Euler transform in SimpleElastix [4–7], allowing spatial information to be related between the two modalities. Time–attenuation curves were then extracted from the internal carotid arteries, describing the variation in image intensity across CTP frames. These curves were characterized by a sharp peak at the time of contrast arrival in the internal carotid and used to identify irrelevant CTP frames before contrast arrival (frames occurring before the main peak) and during secondary cardiac cycles (subsequent lower peaks after the main peak). The internal carotid arteries used for the time–attenuation curves were segmented with an nnU-Net-based model for tubular structure segmentation, using a skeleton-recall loss [8]. After excluding irrelevant frames, the remaining frames were smoothed with a moving-average filter of width three to enforce similar imaging values across neighboring frames. Skull-stripping was performed with TotalSegmentator [9] to restrict the analysis to intracranial regions.

After CTP preprocessing, vessel locations for the time–vessel map were identified from intracranial regions exhibiting high temporal intensity variation across CTP frames. Temporal variation at each location was quantified as the difference between the 90th and 10th image intensity percentiles across frames, normalized by the standard deviation. The resulting variation map was segmented with a k-means estimator in scikit-learn [10] using $k = 3$ to separate vessels from parenchyma and background. Contrast arrival time at each intracranial location was defined as the timestep of maximum intensity, and temporal values were normalized to the range 0.0–1.0 to represent consistent vascular filling patterns across patients. A vessel with a relative contrast-arrival time x would indicate that x% of all intracranial vessels have already received contrast. Lower values in the time–vessel map correspond to arterial filling, while higher values correspond to later venous enhancement.

S1.4. Baseline Detector

The baseline vessel-occlusion detector was trained with the medical object-detection framework nnDetection [11] using a RetinaNet architecture [12], following the configuration proposed by Brugnara et al. [1]. nnDetection automatically optimized preprocessing, anchor sizes, and network depth. Images were resampled to the median spacing of the training data and were normalized by z-scoring after clipping the 0.5 and 99.5 percentiles of all image values in the training set. Detector training used patches of size [96, 160, 160]

and a polynomial learning-rate scheduler starting from an initial learning rate of 0.05 with an SGD optimizer. The loss combined a mean-absolute-error penalty for the estimated occlusion position and a focal loss for occlusion classification. At inference, nnDetection produced bounding boxes with associated confidence scores in the range 0.0–1.0 after non-maximum suppression to remove duplicate or low-confidence detections.

A confidence threshold was determined on an internal validation set of 22 late-phase volumes from the baseline-detector training cohort by maximizing the occlusion-level F2-score, which prioritizes sensitivity over incorrect occlusion detections, since sensitivity is the more critical variable for AIS diagnosis. This threshold was fixed for all subsequent experiments.

S1.5. Post-Processing Correction Framework

The correction framework was applied to detector outputs in late-phase scans at inference time, removing detections inconsistent with anatomical and physiological vascular characteristics derived from time–vessel maps and spatial prior information. Three constraints were applied:

- R1 (anatomical constraint): vessel centerlines were extracted from the vascular regions of the time–vessel map using a skeletonization algorithm in scikit-image [13]. Vessel truncation points were identified as centerline termination points, and detections located beyond a distance threshold from any vessel endpoint were excluded, since estimated occlusions should lie close to points where vessels are interrupted.
- R2 (temporal constraint): detections located in late-enhancing vessels with a contrast-arrival-time value above an estimated threshold were removed, as regions on late-enhancing vessels are inconsistent with arterial occlusions.
- R3 (spatial prior): detections outside a fixed spatial region in the posterior and superior brain were removed. The allowed region was defined from the distribution of annotated occlusions relative to the brain centroid, with an additional fixed margin for robustness, and was fixed prior to external evaluation. The brain centroid was obtained from the TotalSegmentator brain segmentation previously used for skull-stripping.

Constraint parameters were optimized on late-phase cases using vessel-occlusion annotations from the threshold-development subset, minimizing incorrect occlusion detections per scan while preserving occlusion-level sensitivity.

S1.6. CTA-to-Time–Vessel Map Generator

The CTA-to-time–vessel map generator was based on a residual encoder U-Net implemented in nnU-Net [8], operating on skull-stripped brain volumes with Hounsfield Units in intracranial locations and following the default nnU-Net configuration. The architecture included three output heads: a vessel-distance head, a vessel-segmentation head, and a contrast-timing head.

The vessel-distance head was trained to predict Euclidean distance maps to intracranial vessels with an L1 loss. The vessel-segmentation head was trained on binary vessel masks from the ground-truth time–vessel maps using a loss designed for tubular-structure segmentation [14]. The contrast-timing head was trained exclusively on vascular locations from the ground-truth time–vessel maps with an L1 loss. An additional edge-regularization loss was applied across all heads to sharpen spatial predictions [15]:

$$L_{edge} = \frac{1}{\sum_x M(x)} \sum_x M(x) \left| \, ||\nabla P(x)|| \; - \; ||\nabla T(X)|| \, \right|$$

where M(x) denotes the brain mask, ∇P(x) the edges of the predicted map, and ∇T(x) the gradient of the target map.

The generator was trained on the generator training cohort and evaluated on the threshold-development subset. This setup allowed us to compare the performance of the post-processing occlusion removal pipeline when working with CTP-derived time-vessel maps and when working with time-vessel maps from the generator. Consequently, the influence of the CTA-to-time-vessel map generator on the downstream post-processing was analyzed. The evaluation on the threshold-development subset was also used to estimate thresholds for the vessel-distance and segmentation heads. The optimal thresholds corresponded to a vessel-distance threshold of 0.0 mm and a segmentation-logit value of 13.0. The optimized vessel mask was applied to the estimated contrast-timing map to produce the final time–vessel map estimate.

S2. Extended Results

S2.1. Threshold-Development Subset: Parameter Optimization and Sweep

Parameter selection for incorrect-occlusion removal was performed on the 32 late-phase CTA cases with occlusion annotations that form the threshold-development subset. The selected parameters were a distance-to-vessel truncation point of 12 voxels, a relative contrast-arrival time of 0.40, and a 30% margin in the superior and posterior brain regions with respect to the brain centroid. We ran a parameter sweep for different threshold values of the post-processing constraints in the threshold-development subset, finding that the changes in the spatial margin led to the largest performance changes, where taking only 20% of the superior and posterior brain regions with respect to the brain centroid led to true occlusion misses, despite lower incorrect occlusion detections per scan. At the same time, higher normalized time thresholds also increased incorrect occlusion detections per scan, since fewer detections in late-filling vessels were targeted. Results for the parameter sweep are shown in Table S1.

Table S1. Parameter sweep for the constraint thresholds on the threshold-development subset (N = 32). The distance threshold for R1 (in voxels), the relative contrast-arrival-time threshold for R2, and the spatial margin for R3 (as a percentage of the distance from the brain centroid to the brain limits) were varied jointly. Each cell reports the mean with 95% confidence interval (2.5th–97.5th percentile) from bootstrap resampling; values are integers except the incorrect occlusion detections per scan (one decimal). The Baseline row corresponds to the detector without post-processing. The selected configuration (R1 = 12 voxels, R2 = 0.40, R3 = 30%), which minimized incorrect occlusion detections while preserving occlusion-level sensitivity, is shown in bold. FROC, Free-Response Operating Characteristic curve.

Distance R1 (voxels)	Arrival-time R2	Spatial margin R3	FROC	Incorrect occlusion detections / scan	Occlusion sensitivity
Baseline	—	—	46 (33–60)	2.2 (2.1–2.6)	59 (48–65)
8	0.35	20%	50 (36–65)	1.4 (1.3–1.6)	53 (47–59)
8	0.40	20%	51 (36–66)	1.4 (1.2–1.5)	53 (47–59)
8	0.45	20%	50 (35–65)	1.5 (1.4–1.6)	53 (47–59)
12	0.35	20%	50 (35–65)	1.4 (1.3–1.6)	53 (47–59)
12	0.40	20%	51 (36–66)	1.4 (1.2–1.5)	53 (47–59)
12	0.45	20%	50 (35–65)	1.5 (1.4–1.6)	53 (47–59)
16	0.35	20%	50 (35–65)	1.4 (1.3–1.6)	53 (47–59)
16	0.40	20%	51 (36–65)	1.4 (1.2–1.5)	53 (47–59)
16	0.45	20%	50 (35–65)	1.5 (1.4–1.6)	53 (47–59)
8	0.35	30%	49 (36–63)	1.8 (1.6–1.9)	59 (48–65)
8	0.40	30%	49 (35–63)	1.7 (1.4–1.8)	58 (48–65)
8	0.45	30%	47 (34–61)	2.0 (1.8–2.1)	56 (50–62)
12	0.35	30%	49 (36–63)	1.8 (1.6–1.9)	58 (56–62)
**12**	**0.40**	**30%**	**49 (35–63)**	**1.7 (1.4–1.8)**	**59 (48–65)**

Distance R1 (voxels)	Arrival-time R2	Spatial margin R3	FROC	Incorrect occlusion detections / scan	Occlusion sensitivity
12	0.45	30%	47 (34–61)	2.0 (1.8–2.1)	56 (50–62)
16	0.35	30%	49 (36–63)	1.8 (1.7–2.0)	56 (50–62)
16	0.40	30%	49 (35–63)	1.8 (1.7–2.0)	56 (50–62)
16	0.45	30%	47 (34–61)	2.0 (1.8–2.1)	56 (50–62)
8	0.35	40%	49 (36–63)	1.8 (1.7–2.0)	56 (50–62)
8	0.40	40%	49 (35–62)	1.8 (1.7–2.0)	56 (50–62)
8	0.45	40%	47 (34–61)	2.0 (1.8–2.1)	56 (50–62)
12	0.35	40%	49 (35–63)	1.8 (1.7–2.0)	56 (50–62)
12	0.40	40%	49 (35–62)	1.8 (1.7–2.0)	56 (50–62)
12	0.45	40%	47 (34–61)	2.0 (1.8–2.1)	56 (50–62)
16	0.35	40%	49 (35–63)	1.8 (1.7–2.0)	56 (50–62)
16	0.40	40%	48 (35–62)	1.8 (1.7–2.0)	56 (50–62)
16	0.45	40%	47 (34–61)	2.0 (1.8–2.1)	56 (50–62)

S2.2. Patient Population

Additional imaging characteristics for the three cohorts—reconstruction kernel and image dimensions—are summarized in Table S2.

Table S2. Imaging data (reconstruction kernel and image dimensions) for the baseline-detector training, generator training, and external test cohorts. Values are reported as mean (standard deviation). For the generator training cohort, italicized statistics correspond to the late-phase threshold-development subset used for parameter selection. CTA, CT angiography; CTP, CT perfusion.

Characteristic	Detector training	Threshold-development subset	Generator training	External test
Reconstruction kernel	unknown (100%)	H20f (31.2%) / Hr36f (3.1%) / Hr35f (43.7%) / unknown (22%)	H20f (52.9%) / Hr35f (21.5%) / Hr36f (9.0%) / Hv38f (0.4%) / unknown (16.2%)	B (58.5%) / H30f (8.2%) / B25f (3.9%) / B31f (3.9%) / Bv40f (0.6%) / I26f (7.3%) / unknown (17.5%)
Mean CTA dimensions	[512, 512, 647]	[374, 493, 447]	[482, 502, 299]	[513, 515, 576]
Mean CTP dimensions	NA	[512, 512, 137]	[512, 512, 132]	NA

S2.3. External Test Cohort: Per-Center Stratified Results

Per-center results for the late-phase cases of the external test cohort are reported in Tables S3–S6. The best value in each column is shown in bold. Center 3 contained no occlusion-positive cases, so only incorrect occlusion detections per scan are reported for that subset. Given the reduced number of cases per center, the confidence intervals may be wide for certain metrics. Consequently, the center-wise patterns should be considered as indicative rather than definitive.

For Center 1, post-processing correction of the baseline model reduced incorrect occlusion detections per scan from 3.5 (95% CI: 3.1-3.8) to 2.0 (95% CI: 1.8-2.2), increasing FROC from 69 (95% CI: 53-82) to 73 (95% CI: 59-85), AUROC from 82 (95% CI: 72-90) to 85 (95% CI: 76-92), specificity at 95% sensitivity from 19 (95% CI: 6-52) to 32 (95% CI: 13-71), and AUPRC from 65 (95% CI: 47-77) to 70 (95% CI: 55-82). Post-processing correction of the late-phase retraining model also brought improvements, such as the reduction in incorrect occlusion detections per scan from 2.7 (95% CI: 2.4-2.9) to 2.0 (95% CI: 1.8-2.2) or the increase in AUROC from 87 (95%

CI: 78-95) to 90 (95% CI: 82-95). However, the improvements for the late-phase retraining detector were smaller. The detector working with time-vessel maps during training obtained the lowest incorrect occlusion detections per scan overall, 1.3 (95% CI: 1.1-1.5), and the highest AUPRC, 82 (95% CI: 70-91), but also decreased occlusion sensitivity from 77 (95% CI: 63-90) to 74 (95% CI: 59-88).

Table S3. Stratified results in the external test cohort for late-phase cases in Center 1 (Philips only), with 95% confidence intervals. Best values in bold. FROC, Free-Response Operating Characteristic curve; AUROC, area under the Receiver Operating Characteristic curve; AUPRC, area under the Precision-Recall curve.

**Method (*N* = 207, occlusions: 35)**	**Incorrect occlusions / scan**	**FROC**	**Occlusion sensitivity**	**AUROC**	**Specificity at 95% sensitivity**	**AUPRC**
Baseline	3.5 (3.1–3.8)	69 (53–82)	77 (63–90)	82 (72–90)	19 (6–52)	65 (47–77)
Baseline + post-processing correction	2.0 (1.8–2.2)	73 (59–85)	77 (63–90)	85 (76–92)	32 (13–71)	70 (55–82)
Late-phase retraining	2.7 (2.4–2.9)	72 (58–85)	77 (63–90)	87 (78–95)	36 (20–61)	77 (64–89)
Late-phase retraining + post-processing correction	2.0 (1.8–2.2)	**73 (58–85)**	77 (63–90)	**90 (82–95)**	**48 (34–67)**	79 (66–90)
Time–vessel map (extra channel)	**1.3 (1.1–1.5)**	71 (55–84)	74 (59–88)	89 (82–95)	43 (32–56)	**82 (70–91)**

For Center 2, similar patterns were observed, where post-processing reduced the incorrect occlusion detections per scan from 4.6 (95% CI: 3.9-5.3) to 2.1 (95% CI: 1.7-2.5), while increasing FROC 75 (95% CI: 50-94) to 80 (95% CI: 55-98), keeping occlusion sensitivity unchanged at 83 (95% CI: 58-100). However, patient-level metrics degraded, as is the case for AUROC, from 92 (95% CI: 84-98) to 91 (95% CI: 77-100), or specificity at 95% sensitivity, from 66 (95% CI: 46-94) to 55 (95% CI: 27-99). Similar but smaller effects were observed for the late-phase retraining detector, decreasing incorrect occlusion detections per scan from 3.3 (95% CI: 2.8-3.8) to 1.9 (95% CI: 1.6-2.2) at unchanged sensitivity, while specificity at 95% sensitivity decreased from 78 (95% CI: 65-99) to 73 (95% CI: 51-100). The inclusion of time-vessel maps during training provided the lowest incorrect occlusions per scan, 1.7 (95% CI: 1.4-2.1), the highest AUROC, 98 (95% CI: 93-100), the highest specificity at 95% sensitivity, 85 (95% CI: 67-100), and the highest AUPRC, 92 (95% CI: 78-100).

Table S4. Stratified results in the external test cohort for late-phase cases in Center 2 (Siemens only), with 95% confidence intervals. Best values in bold. FROC, Free-Response Operating Characteristic curve; AUROC, area under the Receiver Operating Characteristic curve; AUPRC, area under the Precision-Recall curve.

**Method (*N* = 95, occlusions: 12)**	**Incorrect occlusions / scan**	**FROC**	**Occlusion sensitivity**	**AUROC**	**Specificity at 95% sensitivity**	**AUPRC**
Baseline	4.6 (3.9–5.3)	75 (50–94)	83 (58–100)	92 (84–98)	66 (46–94)	67 (39–89)
Baseline + post-processing correction	2.1 (1.7–2.5)	80 (55–98)	83 (58–100)	91 (77–100)	55 (27–99)	81 (52–98)
Late-phase retraining	3.3 (2.8–3.8)	82 (57–100)	83 (58–100)	95 (89–100)	78 (65–99)	84 (67–99)
Late-phase retraining + post-processing correction	1.9 (1.6–2.2)	**83 (57–100)**	83 (58–100)	95 (87–100)	73 (51–100)	85 (69–100)
Time–vessel map (extra channel)	**1.7 (1.4–2.1)**	**83 (57–100)**	83 (58–100)	**98 (93–100)**	**85 (67–100)**	**92 (78–100)**

For Center 3, only the incorrect occlusions per scan were measured, as all cases were occlusion-negative. In this case, the inclusion of time-vessel maps during training provided the lowest estimate, 1.8 (95% CI: 1.2-2.6). This method was characterized by its aggressiveness against incorrect occlusions. Hence, it was not a complete surprise that it would lead in a center without occlusion-positive cases.

Table S5. Stratified results in the external test cohort for late-phase cases in Center 3 (Siemens only), with 95% confidence intervals. Only incorrect detections per scan are available, since this subset contained no occlusion-positive cases. Best values in bold. FROC, Free-Response Operating Characteristic curve; AUROC, area under the Receiver Operating Characteristic curve; AUPRC, area under the Precision-Recall curve.

**Method (*N* = 17, occlusions: 0)**	**Incorrect occlusions / scan**	**FROC**	**Occlusion sensitivity**	**AUROC**	**Specificity at 95% sensitivity**	**AUPRC**
Baseline	5.6 (4.3–6.9)	—	—	—	—	—
Baseline + post-processing correction	2.6 (1.9–3.4)	—	—	—	—	—
Late-phase retraining	3.2 (2.3–4.2)	—	—	—	—	—
Late-phase retraining + post-processing correction	1.9 (1.2–2.5)	—	—	—	—	—
Time–vessel map (extra channel)	**1.8 (1.2–2.6)**	—	—	—	—	—

Similar patterns to centers 1 and 2 were observed in center 4, where post-processing correction of the baseline detector reduced the incorrect occlusion detections per scan from 3.4 (95% CI: 2.7-4.1) to 2.0 (95% CI: 1.5-2.5). For the late-phase retraining detector, the incorrect occlusion detections per scan decreased from 2.5 (95% CI: 1.9-3.1) to 1.7 (95% CI: 1.2-2.2). Training with time-vessel maps as an extra channel reduced incorrect occlusions per scan down to 1.3 (95% CI: 0.9-1.8), but decreased FROC to 90 (95% CI: 57-100), AUROC to 88 (95% CI: 50-100), and AUPRC to 80 (95% CI: 7-100).

Table S6. Stratified results in the external test cohort for late-phase cases in Center 4 (Siemens only), with 95% confidence intervals. Best values in bold. FROC, Free-Response Operating Characteristic curve; AUROC, area under the Receiver Operating Characteristic curve; AUPRC, area under the Precision-Recall curve.

**Method (*N* = 36, occlusions: 4)**	**Incorrect occlusions / scan**	**FROC**	**Occlusion sensitivity**	**AUROC**	**Specificity at 95% sensitivity**	**AUPRC**
Baseline	3.4 (2.7–4.1)	99 (86–100)	99 (100–100)	100 (100–100)	100 (100–100)	**100 (100–100)**
Baseline + post-processing correction	2.0 (1.5–2.5)	99 (86–100)	99 (100–100)	99 (100–100)	100 (100–100)	**100 (100–100)**
Late-phase retraining	2.5 (1.9–3.1)	**100 (100–100)**	99 (100–100)	99 (100–100)	100 (100–100)	**100 (100–100)**
Late-phase retraining + post-processing correction	1.7 (1.2–2.2)	**100 (100–100)**	99 (100–100)	100 (100–100)	100 (100–100)	**99 (100–100)**
Time–vessel map (extra channel)	**1.3 (0.9–1.8)**	90 (57–100)	99 (100–100)	88 (50–100)	69 (34–100)	80 (7–100)

S2.4. CTA-to-Time–Vessel Map Generator

To study the fidelity of the CTA-to-time-vessel map generator, the generated time-vessel maps in the threshold-development subset were compared with the CTP-derived maps. For vessel segmentation, a Dice coefficient of 60 (95% CI: 58-62) was obtained. For vessel distance estimation and time estimation, Mean Absolute Errors of 2.28 (95% CI: 2.05-2.53) mm and 0.29 (95% CI: 0.27-0.31) were obtained, respectively. These values are also included in Table S7.

Table S7. Quantitative results for the CTA-to-time–vessel map generator on the test partition of the generator training cohort (N = 51). Values are point estimates with 95% confidence intervals. MAE, mean absolute error.

**Metric**	**Value**
Dice coefficient	60 (58–62)
MAE, normalized time error	0.29 (0.27–0.31)
MAE, distance error (mm)	2.28 (2.05–2.53)

S2.5. Additional baseline performance in threshold-development subset

The additional detectors ("late-phase retraining" and "time-vessel map (extra channel)") were also evaluated in the threshold-development subset (Supplementary Table S8 and Supplementary Figure S1). Post-processing correction improved results for the late-phase retraining detector, although with a slight decrease in occlusion sensitivity in this specific subset, whereas supplying time-vessel maps during detector training improved FROC and occlusion sensitivity, but increased the number of incorrect occlusions per scan.

Table S8. Vessel occlusion detection performance on the threshold-development subset (quantitative results).

**Method (N=32, occlusions: 32)**	**FROC**	**Incorrect occlusion detections / scan**	**Occlusion sensitivity**
Baseline	46 (33–60)	2.3 (2.1–2.6)	58 (48–65)
Baseline + post-processing correction	49 (35–63)	**1.7 (1.4–1.8)**	58 (48–65)
Late-phase retraining	60 (44–77)	3.0 (2.4–3.7)	**72 (56–88)**
Late-phase retraining + post-processing correction	**60 (43–78)**	2.5 (1.9–3.1)	69 (53–84)
Time vessel map (extra channel)	52 (37-67)	2.0 (1.4-2.6)	62 (46-78)

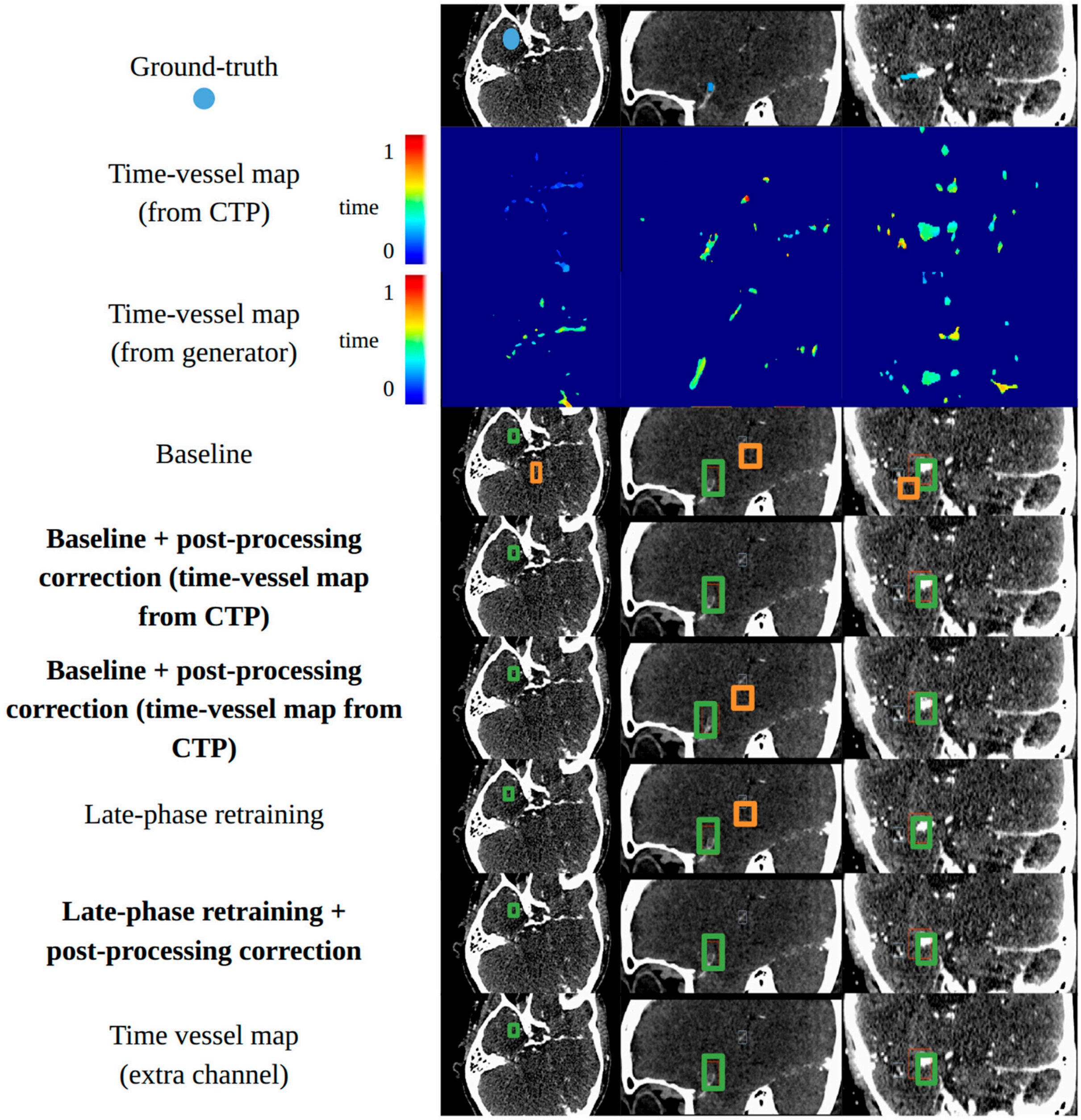

Figure S1. Detection performance on the threshold-development subset (qualitative results), including baseline post-processing with CTP-derived time-vessel maps and baseline post-processing with generator-derived time-vessel maps. Blue blobs denote vessel-occlusion annotations, orange boxes denote incorrect occlusion detections, and green boxes denote true occlusion detections.

References

1. Brugnara, G.; Baumgartner, M.; Scholze, E.D.; Deike-Hofmann, K.; Kades, K.; Scherer, J.; Denner, S.; Meredig, H.; Rastogi, A.; Mahmutoglu, M.A.; et al. Deep-Learning Based Detection of Vessel Occlusions on CT-Angiography in Patients with Suspected Acute Ischemic Stroke. *Nat. Commun.* **2023**, *14*, 4938, doi:10.1038/s41467-023-40564-8.
2. Mulder, M.J.H.L.; Jansen, I.G.H.; Goldhoorn, R.-J.B.; Venema, E.; Chalos, V.; Compagne, K.C.J.; Roozenbeek, B.; Lingsma, H.F.; Schonewille, W.J.; van den Wijngaard, I.R.; et al. Time to Endovascular Treatment and Outcome in Acute Ischemic Stroke: MR CLEAN Registry Results. *Circulation* **2018**, *138*, 232–240, doi:10.1161/CIRCULATIONAHA.117.032600.
3. Rodriguez-Luna, D.; Dowlatshahi, D.; Aviv, R.I.; Molina, C.A.; Silva, Y.; Dzialowski, I.; Lum, C.; Czlonkowska, A.; Boulanger, J.-M.; Kase, C.S.; et al. Venous Phase of Computed Tomography Angiography Increases Spot Sign Detection, but Intracerebral Hemorrhage Expansion Is Greater in Spot Signs Detected in Arterial Phase. *Stroke* **2014**, *45*, 734–739, doi:10.1161/STROKEAHA.113.003007.

4. Marstal, K.; Berendsen, F.; Staring, M.; Klein, S. SimpleElastix: A User-Friendly, Multi-Lingual Library for Medical Image Registration. In Proceedings of the 2016 IEEE Conference on Computer Vision and Pattern Recognition Workshops (CVPRW); June 2016; pp. 574–582.
5. Beare, R.; Lowekamp, B.; Yaniv, Z. Image Segmentation, Registration and Characterization in R with SimpleITK. *J. Stat. Softw.* **2018**, *86*, 8, doi:10.18637/jss.v086.i08.
6. Yaniv, Z.; Lowekamp, B.C.; Johnson, H.J.; Beare, R. SimpleITK Image-Analysis Notebooks: A Collaborative Environment for Education and Reproducible Research. *J. Digit. Imaging* **2018**, *31*, 290–303, doi:10.1007/s10278-017-0037-8.
7. Lowekamp, B.C.; Chen, D.T.; Ibáñez, L.; Blezek, D. The Design of SimpleITK. *Front. Neuroinformatics* **2013**, *7*, 45, doi:10.3389/fninf.2013.00045.
8. Isensee, F.; Jaeger, P.F.; Kohl, S.A.A.; Petersen, J.; Maier-Hein, K.H. nnU-Net: A Self-Configuring Method for Deep Learning-Based Biomedical Image Segmentation. *Nat. Methods* **2021**, *18*, 203–211, doi:10.1038/s41592-020-01008-z.
9. Wasserthal, J.; Breit, H.-C.; Meyer, M.T.; Pradella, M.; Hinck, D.; Sauter, A.W.; Heye, T.; Boll, D.T.; Cyriac, J.; Yang, S.; et al. TotalSegmentator: Robust Segmentation of 104 Anatomic Structures in CT Images. *Radiol. Artif. Intell.* **2023**, *5*, e230024, doi:10.1148/ryai.230024.
10. Pedregosa, F.; Varoquaux, G.; Gramfort, A.; Michel, V.; Thirion, B.; Grisel, O.; Blondel, M.; Prettenhofer, P.; Weiss, R.; Dubourg, V.; et al. Scikit-Learn: Machine Learning in Python. *J Mach Learn Res* **2011**, *12*, 2825–2830.
11. Baumgartner, M.; Jäger, P.F.; Isensee, F.; Maier-Hein, K.H. nnDetection: A Self-Configuring Method for Medical Object Detection. In Proceedings of the Medical Image Computing and Computer Assisted Intervention – MICCAI 2021; de Bruijne, M., Cattin, P.C., Cotin, S., Padoy, N., Speidel, S., Zheng, Y., Essert, C., Eds.; Springer International Publishing: Cham, 2021; pp. 530–539.
12. Lin, T.-Y.; Goyal, P.; Girshick, R.; He, K.; Dollár, P. Focal Loss for Dense Object Detection 2018.
13. Walt, S.V.; Schönberger, J.L.; Nunez-Iglesias, J.; Boulogne, F.; Warner, J.D.; Yager, N.; Gouillart, E.; Yu, T.; contributors, the scikit-image Scikit-Image: Image Processing in Python. *PeerJ* **2014**, doi:10.7717/peerj.453.
14. Kirchhoff, Y.; Rokuss, M.R.; Roy, S.; Kovacs, B.; Ulrich, C.; Wald, T.; Zenk, M.; Vollmuth, P.; Kleesiek, J.; Isensee, F.; et al. Skeleton Recall Loss for Connectivity Conserving and Resource Efficient Segmentation of Thin Tubular Structures 2024.
15. Hong, J.P.; Cho, S.J.; Lee, J.; Ji, S.W.; Ko, S.J. Single Image Deblurring Based on Auxiliary Sobel Loss Function: 2020 IEEE International Conference on Consumer Electronics - Asia, ICCE-Asia 2020. *2020 IEEE Int. Conf. Consum. Electron. - Asia ICCE-Asia 2020* **2020**, doi:10.1109/ICCE-Asia49877.2020.9276836.
